# Global Lysine Crotonylation and 2-Hydroxyisobutyrylation in Phenotypically Different *Toxoplasma gondii* Parasites*[Fn FN2]

**DOI:** 10.1074/mcp.RA119.001611

**Published:** 2019-09-05

**Authors:** Deqi Yin, Ning Jiang, Yue Zhang, Dawei Wang, Xiaoyu Sang, Ying Feng, Rang Chen, Xinyi Wang, Na Yang, Qijun Chen

**Affiliations:** ‡ Key Laoratory of Animal Infectious Diseases in Northeast China, Ministry of Education, Key Laboratory of Zoonosis, Shenyang Agricultural University, 120 Dongling Road, Shenyang 110166, China; § College of Basic Education, Shenyang Agricultural University, 120 Dongling Road, Shenyang 110166, China

**Keywords:** Parasite, proteogenomics, virulence, pathway analysis, protein-protein interactions, phenotype, posttranslational modification, proteome, regulation, Toxoplasma gondii

## Abstract

*Toxoplasma gondii* is a unicellular protozoan parasite of the phylum Apicomplexa. The parasite repeatedly goes through a cycle of invasion, division and induction of host cell rupture, which is an obligatory process for proliferation inside warm-blooded animals. It is known that the biology of the parasite is controlled by a variety of mechanisms ranging from genomic to epigenetic to transcriptional regulation. In this study, we investigated the global protein posttranslational lysine crotonylation and 2-hydroxyisobutyrylation of two *T. gondii* strains, RH and ME49, which represent distinct phenotypes for proliferation and pathogenicity in the host. Proteins with differential expression and modification patterns associated with parasite phenotypes were identified. Many proteins in *T. gondii* were crotonylated and 2-hydroxyisobutyrylated, and they were localized in diverse subcellular compartments involved in a wide variety of cellular functions such as motility, host invasion, metabolism and epigenetic gene regulation. These findings suggest that lysine crotonylation and 2-hydroxyisobutyrylation are ubiquitous throughout the *T. gondii* proteome, regulating critical functions of the modified proteins. These data provide a basis for identifying important proteins associated with parasite development and pathogenicity.

## Abstract (precis)

The proteomes of the extracellular stage of the two phenotypically different *Toxoplasma gondii* strains, RH strain and ME49 strain, were systematically analyzed using an LC-MS/MS approach, and the global lysine crotonylation and 2-hydroxyisobutyrylation of the two parasite strains were deeply investigated. The global maps of lysine crotonylation and 2-hydroxyisobutyrylation of the proteomes of the two parasite strains will provide a valuable resource to facilitate the illumination of the biology of the zoonotic *T. gondii* parasite.

## Graphical Abstract

**Figure:**
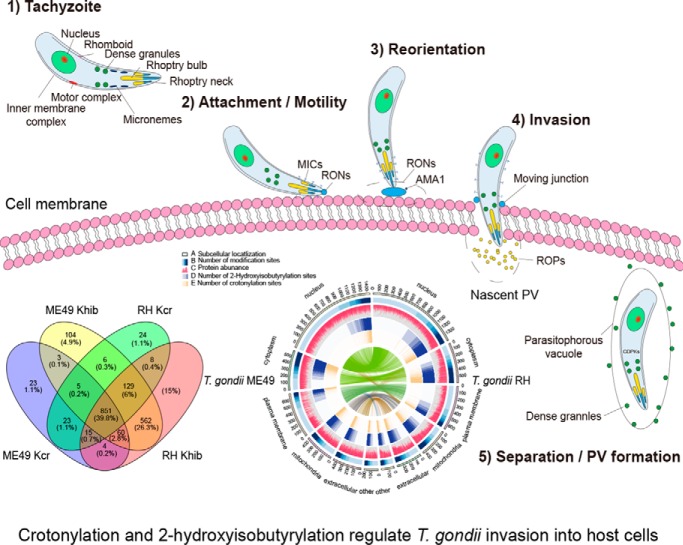

**Highlights**

- Quantitative proteomes and epigenetic regulation of *T. gondii*.
- Protein crotonylation and 2-hydroxyisobutylation in phenotypically different *T. gondii* parasites.
- Regulation of invasion regulation of *T. gondii* by protein modification.
- Lysine crotonylation and 2-hydroxyisobutylation regulated in multiple biological processes in phenotypically different *T. gondii* parasites.

*Toxoplasma gondii* is an intracellular parasite that infects all warm-blooded animals and is disseminated frequently via contaminated meat (1, 2). Toxoplasmosis is one of the most prevalent zoonoses worldwide. Feline animals, particularly cats, are the definitive hosts of the parasite and where *T. gondii* undergoes sexual reproduction within the intestine epithelial cells, and millions of oocysts are shed in the faeces of infected cats. Animals and human beings are primarily infected by ingestion of oocyst-contaminated water and feed or cyst-contaminated meat. In infected animals, the parasites invade nucleated cells such as macrophages, dendritic cells (DCs)^1^ and muscle cells. *T. gondii* poses a great threat to individuals whose immune systems are compromised, such as patients with HIV infections or those receiving immune suppression treatment (3). The direct damage caused by the parasites is massive cell lysis and tissue dysfunction. The parasite can traverse the placenta and proliferate in the fetus, making it one of the pathogens threatening the health of pregnant women and newborns (4).

After invasion into a host cell, *T. gondii* replicates into several daughter cells named tachyzoites, which rapidly invade new host cells after egress. In immune-competent hosts, the parasites convert into a semidormant state with slow development, named bradyzoites, in tissue cysts, which can be activated immediately if the host immune capacity is weakened. Currently, effective drugs of choice against *T. gondii* are limited to a few metabolic inhibitors such as pyrimethamine, sulfonamides, spiramycin, and clindamycin (5). They are effective in only replicative tachyzoites, with little or no effect on the semidormant bradyzoites, and some are quite cytotoxic to the hosts. The discovery of new drug targets is essential but relies on a deep understanding of parasite biology.

The genome of *T. gondii* is ∼70 Mb, encoding more than 7000 proteins, which may be expressed at distinct developmental stages in various quantities (6) (www.ToxoDB.org). As in other organisms, a majority of *T. gondii* proteins are localized in functionally specialized organelles, such as food vacuoles, rhoptries, dense granules and micronemes. Further, *T. gondii* proteins are also post-translationally modified to achieve their functionality. Post-translational modification (PTM) is a process in which a chemical moiety is covalently added to certain amino acid groups after a protein is translated from its mRNA template. These modifications include acetylation, glycosylation, ubiquitination, nitrosylation, methylation, phosphorylation, and lipidation, which influence almost all aspects of cell biology and pathogenesis. Therefore, identification of and understanding the mechanisms of PTMs is critical in the study of cell function and disease treatment and prevention (7, 8). In recent years, PTM studies in parasitology have been on mainly *Plasmodium falciparum*, the causative agent of malaria, and *T. gondii* (8, 9). In *T. gondii*, earlier studies suggested that methylation, acetylation, ubiquitylation, succinylation, SUMOylation and glycosylation were widely distributed in the proteome of the parasite (8). Several key proteins, such as TgGAP40, TgGAP45, TgGAP50, and TgMLC1, involved in tachyzoite gliding motility, moving junction (MJ) formation, and other functions that are essential for host-cell invasion are modified by ubiquitination, phosphorylation and N-glycosylation (8, 10, 11). These modifications obviously regulate parasite movement by affecting protein distribution or polymerization. With FLAG-affinity chromatography techniques, 120 proteins in the categories of chromatin and transcriptional machinery, ribosomal biogenesis, translation-related proteins (ROPs), stress-related proteins and bradyzoite parasitophorous vacuole membrane proteins were found to be SUMOylated (12). Recently, with the application of high-throughput mass spectrometry, histone modifications, “the histone code” and core markers of epigenetic regulation, of several protozoan parasites have been gradually dissected (9). To date, chromatin modifications are the most well-studied PTMs in most organisms. Histone modifications that affect gene activation and suppression include methylation, acetylation, ubiquitination, glycosylation, phosphorylation, ADP-ribosylation and SUMOylation, which frequently occur at the N-terminal tails of histone units (9). The functions of many histone PTMs appear to be evolutionarily conserved in the Apicomplexan parasite. In general, histone acetylation is associated with gene activation, whereas methylation can be associated with either gene repression or activation depending upon the residues modified. In *T. gondii*, the most conserved modification is trimethylation of histone 3 lysine 4 (H3K4me3), which is a marker of transcriptionally active promoters. Further, it is likely that the classic gene activation marks H3K4me3, H4ac, and H3K9ac are co-localized and mark the promoters of actively transcribed genes in euchromatins of *T. gondii* (13). Apart from histone modifications, PTMs also occur in proteins of other cellular structures. Xiao *et al.* found that acetylation, polyglutamylation, and methylation were present in the C-terminal alpha- and beta-tubulin of *T. gondii* (14).

Although strains of the type I (such as the RH strain) are virulent and uniformly lethal to mice, causing severe clinical manifestations of toxoplasmosis, strains of the types II (such as the ME49 strain) are relatively low virulence to murine hosts, which are able to control acute phase of the disease and are able to establish chronic infections (15, 16). Thus, the two strains provide ideal models for study genetic and epigenetic regulations in parasite biology. In this study, the proteomes of the extracellular stage of the two phenotypically different *T. gondii* strains, RH strain and ME49 strain, were systematically analyzed using an LC-MS/MS approach, and the global lysine crotonylation and 2-hydroxyisobutyrylation of the two parasite strains were deeply investigated.

## EXPERIMENTAL PROCEDURES

### 

#### 

##### Experimental Design and Statistical Rationale

Global proteome, crotonylation and 2-hydroxyisobutyrylation of soluble proteins derived from phenotypically different *T. gondii* parasites (RH and ME49 strains) which were analyzed using label-free mass spectrometry. Three biological replicates of the two *T. gondii* strains were analyzed in each experiment in order to validate the biological reliability of measurements. Qualitative analysis and quantitative analysis were respectively carried out in different virulent strains. We applied a standard *t* test to determine if there is a statistically significant difference between the two *T. gondii* strains. Statistical Testing and generation of graphs was performed with R.

##### Parasite Culture and Protein Extraction

Tachyzoites of the *T. gondii* RH and ME49 strains were purified from peritoneal fluid by passing through 5.0 μm Nucleopore filters and Percoll gradient centrifugation (GE Healthcare, Uppsala, Sweden). The parasite cells were washed using cold, sterile phosphate-buffered saline (PBS-1×). After addition of lysis buffer (1% protease inhibitor mixture, 8 m urea) to purified *T. gondii* cells, the cells were then sonicated three times on ice with a high-intensity sonicator (Scientz, Ningbo, China). To separate insoluble fragments, the lysate was centrifuged at 12,000 × *g* at 4 °C for 10 mins. Eventually, the supernatant in the centrifuge tube was collected, and the protein concentration was assayed using a BCA kit (Beyotime, Shanghai, China).

##### Trypsin Digestion and HPLC Fractionation

Before trypsinization, dithiothreitol was added to the protein solution to a final concentration of 5 mm, and the solution was reduced at 56 °C for 30 mins; then, 11 mm iodoacetamide (Sigma, Saint Louis, MO) was used to alkylate the proteins for 15 mins at 37 °C in a darkroom. The urea concentration of the sample was diluted to less than 2 m with a 100 mm_NH4HCO3_ solution. Trypsin was added at a mass ratio of 1:50 (pancreatin/protein) for the first time and digested overnight. Then, trypsin was added again at a mass ratio of 1:100 (pancreatin/protein) and digested for 4 h. The tryptic peptides were separated by high-pH reversed-phase fractionation (RPF) with a Thermo Betasil C18 column (5 μm particles, 10 mm ID, 250 mm length). In the final step, peptides were divided into 60 fractions within 60 mins with a concentration gradient of 8% to 32% acetonitrile (pH 9.0) and then further divided into 4 fractions. Afterward, the peptides were subjected to vacuum freeze drying for in-depth analysis.

##### Antibody-based PTM Enrichment

The peptides were dissolved in an immunoprecipitation (IP) buffer solution (100 mm NaCl, 1 mm EDTA, 50 mm Tris-HCl, 0.5% NP-40, pH 8.0), and the supernatant was transferred to a pre-washed dihydroxyisobutyrylated and crotonylated resin (PTM-804 and PTM-502, Hangzhou, China). Then, the solution was placed on a rotary shaker at 4 °C, gently shaken and incubated overnight. After incubation, the resin was washed 4 times with the IP buffer solution and twice with deionized water. Finally, the resin-bound peptides were eluted three times with 0.1% trifluoroacetic acid eluate, and the eluate was collected and vacuum dried. After draining, a desalting operation was carried out according to the C18 ZipTips instructions, and the peptides were vacuum dried and prepared for LC-MS/MS analysis.

##### LC-MS/MS Analysis

The peptides were dissolved in liquid phase A (0.1% (v/v) aqueous formic acid) and separated using an EASY-nLC 1000 ultra-performance liquid chromatography (UPLC) system. Solvent B (0.1% formic acid in 98% acetonitrile) was run on the EASY-nLC 1000 UPLC system, maintaining a flow rate of 400 nL/min, and the gradient was increased from 6% to 23% in 26 mins, increased from 23% to 35% in 8 mins and increased to 80% in 3 mins, then maintained at 80% for the last 3 mins. Using UPLC to separate the peptides, they were ionized by a nanospray ionization (NSI) ion source (voltage was 2.0 kV) and finally analyzed using Q ExactiveTM HF-X mass spectrometry. The primary mass spectrometer scan range was 350–1,600 *m*/*z* (scan resolution set to 60,000), and the secondary scan resolution was up to 15,000. The top 20 peptides with the highest signal intensity were selected by the data-dependent scan (DDA) program to enter the higher-energy collisional dissociation (HCD) cells and then fragmented using 28% fragmentation energy after the first scan. Then, secondary mass spectrometry was performed in sequence. Automatic gain control (AGC) was set to 5E4.

##### Database Search

Secondary mass spectral data (repeated in three experiments) were retrieved using MaxQuant (v1.5.2.8). Tandem mass spectra were searched against the UniProtKB *Toxoplasma gondii* (strain ATCC 50611/Me49) database (ToxoDB 36, 8,315 sequences) concatenated with a reverse decoy database, and an anti-library was added to calculate the false positive rate (FDR) caused by random matching. The enzyme digestion mode was set to Trypsin/P, which allowed up to 4 missing cleavages. The mass error tolerances of the main search and first search were set to 5 ppm and 20 ppm, respectively. The mass error tolerance of the secondary fragment ions was 0.02 Da. For identification of crotonylation, carbamidomethyl on cysteine (Cys) was specified as fixed modification and lysine (Lys) crotonylation modification, oxidation on methionine (Met) and acetylation on protein N-terminal were specified as variable modifications. For identification of 2-hydroxyisobutylation, carbamidomethyl on Cys was specified as fixed modification and Lys 2-hydroxyisobutyrylation modification, oxidation on Met and acetylation on protein N-terminal were specified as variable modifications. The label-free quantification method was LFQ, FDR was set to < 1%, and the lowest score of modified peptides was adjusted to > 40.

##### Western Blotting

Protein lysate (20 μg) generated from the tachyzoites of both the RH and ME49 *T. gondii* strains was separated using 12% Bis-Tris polyacrylamide gels and then transferred to nitrocellulose filter membranes (Bio-Rad, Hercules, CA). Next, 5% skim milk using Tris-buffered saline solution with Tween (TBST) was used to block the membrane for 1 hour at 37 °C, and then the membrane was incubated with antibodies to crotonyllysine (Cat#: PTM-502) (1: 2000; PTM BIO) and 2-hydroxybutyryllysine (Cat#: PTM-801) (1: 1000; PTM BIO) at 4 °C overnight. The membrane was washed three times using TBST buffer before incubation with a secondary antibody at 37 °C for 1 hour (1:10,000; Thermo Scientific™ Pierce, 31430, 31460, MA).

##### Immunofluorescence

To detect modified proteins in the parasites, the tachyzoites of the *T. gondii* RH and *T. gondii* ME49 strains were fixed on slides using pre-chilled paraformaldehyde for 20 mins. The parasites were permeabilized by 0.1% Triton X-100 for 20 mins at room temperature. First, the slides were blocked with 5% skim milk for 1 hour at 37 °C and then incubated with primary antibodies recognizing crotonyllysine (Cat#: PTM-502) and 2-hydroxybutyryllysine (Cat#: PTM-801) (PTM BIO) for 1 hour at 37 °C. Then, the cells were incubated for 30 min at 37 °C using a fluorescent secondary antibody (Thermo, MA), and the nuclei were stained as previously described (17, 18). High-resolution images were captured by a confocal laser scanning microscope (Leica, SP8, Wetzlar, Germany).

### Bioinformatics Methods

#### 

##### Quantitative Method

Intensity of peptides in each sample was performed based on MS peak area, and the intensity of each protein was calculated by MaxQuant LFQ method. For PTM sites, intensity was sum of all the modified peptides containing this PTM site. The relative quantification of each sample was obtained based on the protein (PTM sites) intensity between different samples. Three replicates were performed in this study. The average intensity of three replicates was calculated to represent the overall intensity of the protein (PTM sites) in the sample. Two-sample two-tailed *t* test method was used to calculate *p* value of difference abundance in protein and PTM sites level.

##### Quantitative Analysis

The spectra were first analyzed by MaxQuant software, and the proteome and modified groups were quantified using label-free quantitation. The quantitative value of RH/ME49 is the average of three replicate quantitative values. The difference significance (*p* value) was calculated by *t* test. When RH/ME49 had a fold change > 1.2 and *p* < 0.05, it was a differential protein. When RH/ME49 had a fold change >1.5 and *p* < 0.05, it was a differential modification site. When a protein was identified in three replicates of the RH strain and in none of the three replicates of the ME49 strain, it was an RH-specific expressed protein or a specific modification site. In the opposite scenario, it was a specific modification site of the ME49 strain.

##### GO Annotation

GO annotations were based on the UniProt-GOA database (www.http://www.ebi.ac.uk/GOA/). The identified protein IDs were matched and converted to the ID of the UniProt database and then mapped to the GO ID using the protein ID. When some of the identified proteins were not annotated with the UniProt-GOA database, the remaining proteins were annotated based on the protein sequence alignment method using InterProScan software. Proteins were classified according to the following three areas of GO annotation: cellular component, molecular function, and biological process.

##### KEGG Pathway Annotation

The KEGG database was used to annotate the proteins of relevant pathways. First, the proteins were annotated via the KEGG online service tool KAAS, and they were matched into the corresponding pathways using the KEGG mapper.

##### KOG Annotation

The KOG annotation proteome was derived from the NCBI-COG database (https://www.ncbi.nlm.nih.gov/COG/). The sequences of differentially modified proteins were matched to the basic local alignment search tool (BLAST) version 2.2.28 KOG database to obtain protein KOG annotation information.

##### Subcellular Localization

Subcellular localization annotation of the submitted proteins was performed using the software wolfpsort (a new version of PSORT/PSORT II), which can be used to predict the subcellular localization of eukaryotic sequences.

##### Motif Analysis

Motif-x software was used to analyze the motif features of the modified sites. Comparative analysis of a modified 21-mer sequence model consisting of all identified modification sites (ten sites upstream and ten sites downstream) was performed. When the number of peptides in a certain characteristic sequence was > 20 and the statistical test *p* value was < 0.000001, it was a motif of the modified peptides.

##### Enrichment of GO Analysis

For every category, a two-tailed Fisher's exact test was used to test the extent to which differentially modified proteins were enriched for all identified proteins according to the GO function annotation. In addition, a corrected *p* value < 0.05 was significantly enriched.

##### Enrichment of Pathways

The enrichment pathways were identified by a two-tailed Fisher exact test using the KEGG database to test the enrichment of differentially modified proteins for all identified proteins. If the corrected *p* value of the pathway enrichment was < 0.05, it was considered significant.

##### Functional Enrichment Heatmap

All functional enriched *p* values were collected and then screened for functional classifications that were significantly enriched (*p* value < 0.05) in at least one group. Then, the obtained *p* value data matrix was first subjected to logarithmic transformation with -log10, which was subjected to Z transform for each function classification. Finally, the data were analyzed by hierarchical clustering (Euclidean distance, average-linkage clustering). The clustering relationship was visualized via the function pheatmap in the R language package.

##### Protein-Protein Interaction Network Analysis

The network of protein-protein interactions was obtained from STRING database (19) and visualized using Cytoscape software (20).

## RESULTS

### 

#### 

##### Numerous Proteins Differentially Expressed in the Two T. gondii Strains Were Identified

**Fig. 1.**
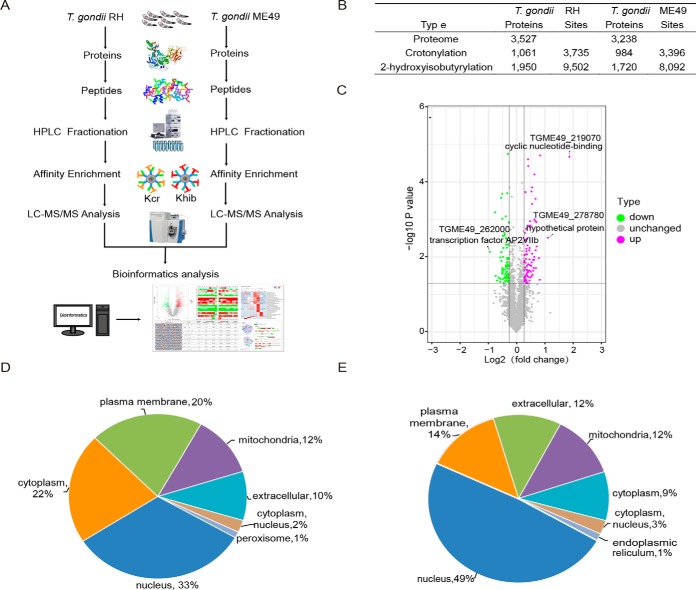
**Quantitative analysis differences in proteomes between the *T. gondii* RH strain and the ME49 strain.** *A*, Schematic illustration of the proteomic analytical steps for protein purification, trypsinization, antibody-based enrichment, and bioinformatic analysis after LC-MS/MS. *B*, Summary of qualitative data identified in the phenotypically different *T. gondii* strains (supplemental Data S1, S2). *C*, Volcano plot of differentially expressed proteins in the two *T. gondii* strains. Magenta dots represent upregulated proteins in the *T. gondii* RH strain (RH/ME49 ratio > 1.2; *t* test, *p* < 0.05). Green dots represent upregulated proteins in the *T. gondii* ME49 strain (RH/ME49 ratio < 1/1.2; *t* test, *p* < 0.05). Detailed data are listed in supplemental Data S3. *D*, Pie charts showing upregulated proteins classified by localization in the *T. gondii* RH strain, excluding unknown proteins. Detailed data are listed in supplemental Data S4. *E*, Pie charts showing upregulated proteins classified by localization in the *T. gondii* ME49 strain, excluding unknown proteins. Detailed data are listed in supplemental Data S4.

The soluble proteins obtained from both the *T. gondii* RH and ME49 strains were separately analyzed for LC-MS/MS identification with 3 replications (Fig. 1*A*). Pearson correlation of Log2 LFQ intensity between replications was 1, and 75% coefficient variation of three replications was less than 0.01 (supplemental Fig. S1). In total, 3527 and 3238 proteins were identified in the *T. gondii* RH and ME49 strains, respectively (Fig. 1*B*). A total of 2,855 proteins contained quantitative information (supplemental Data S3). Of these, 84 proteins were upregulated in the *T. gondii* RH strain, and 74 were upregulated in the *T. gondii* ME49 strain (Fig. 1*C*). The differentially expressed proteins between the two *T. gondii* strains were in mainly the nucleus, cytoplasm, plasma membrane, and mitochondria (Fig. 1*D* and 1*E*). The 84 upregulated proteins in RH strain *T. gondii* were significantly enriched in the endoplasmic reticulum, with transferase and oxidoreductase activities. In contrast, in the ME49 strain, the upregulated proteins were more diverse, ranging from ribosomal complex proteins to membrane bound and unbound proteins, which were highly enriched in functions associated with protein dimerization activity and organonitrogen biosynthetic process (supplemental Data S4).

##### Global Lysine Crotonylation and 2-Hydroxyisobutyrylation of T. gondii Proteins

**Fig. 2.**
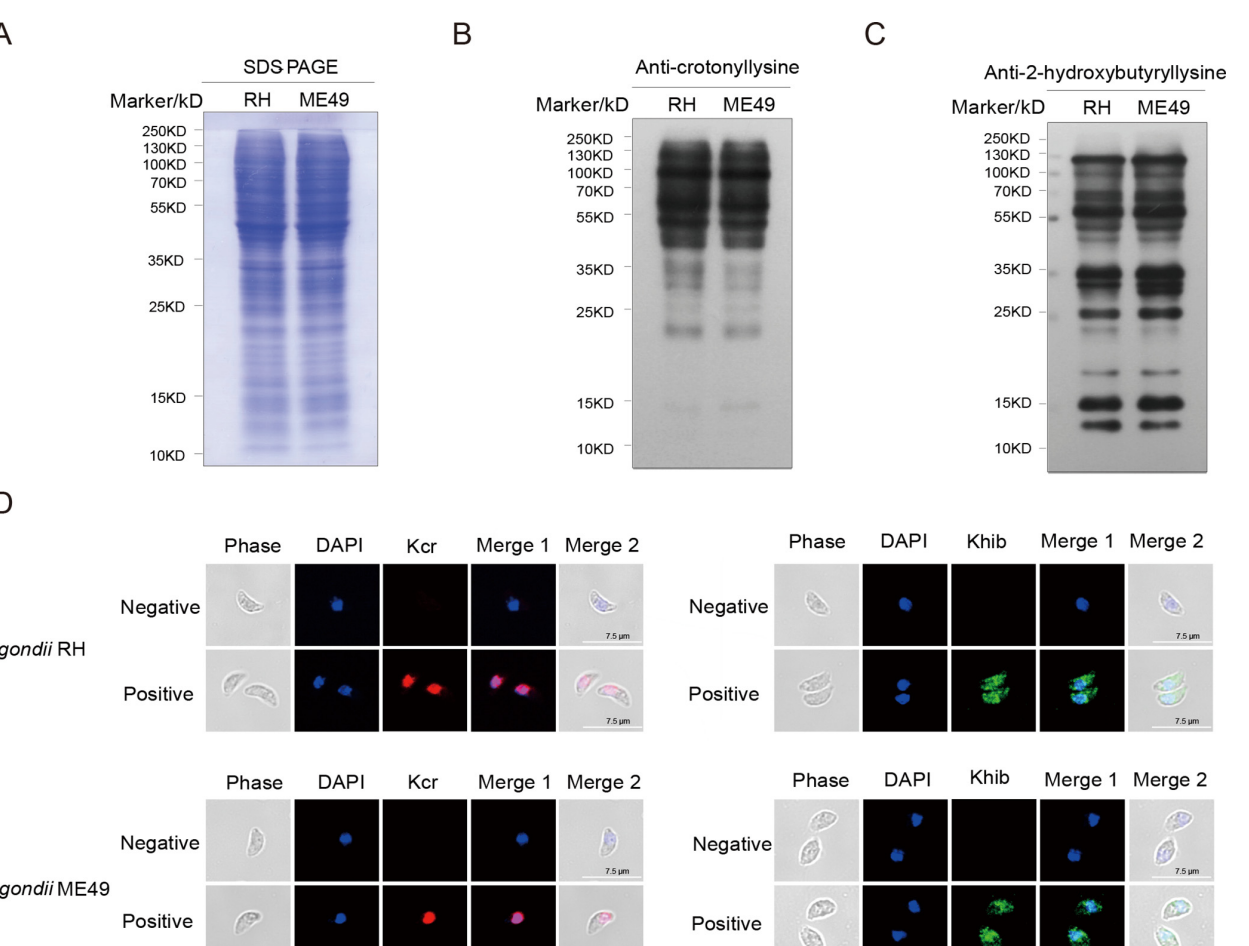
**Western blotting and immunofluorescence analysis of lysine crotonylation and 2-hydroxyisobutyrylation in *T. gondii* strains.** *A*, SDS-PAGE analysis of tachyzoite lysates of the *T. gondii* RH and ME49 strains. *B*, and *C*, Western blot analysis of 20 μg of tachyzoite lysate probed with anti-crotonyllysine and anti-2-hydroxybutyryllysine antibodies. *D*, Immunofluorescence staining of paraformaldehyde-fixed tachyzoites of *T. gondii* RH and *T. gondii* ME49 with an anti-crotonyllysine antibody (red). Immunofluorescence staining of pre-chilled paraformaldehyde-fixed tachyzoites of *T. gondii* RH and *T. gondii* ME49 with an anti-2-hydroxybutyryllysine antibody (green). Nuclei were stained with DAPI (blue).

Protein modification in tachyzoites was confirmed by Western blotting and immunofluorescence assay (IFA) with anti-crotonyllysine and anti-2-hydroxybutyryllysine antibodies (Fig. 2*A*–2*C*). The proteins with 2-hydroxybutyryllysines were widely distributed inside multiple discrete compartments of the two *T. gondii* strains, whereas proteins with crotonyllysines were abundant in the nucleus (Fig. 2*D* and 2*E*).

**Fig. 3.**
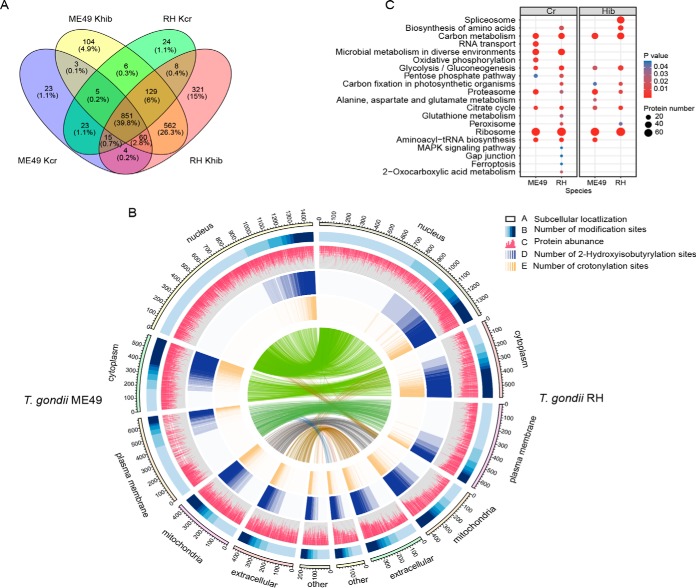
**Qualitative analysis of lysine crotonylation and 2-hydroxyisobutyrylation in *T. gondii*.** *A*, Venn diagram showing overlap between lysine crotonylated and 2-hydroxyisobutyrylated proteins in *T. gondii* strains. Ellipses in green and pink represent the numbers of crotonylated proteins and 2-hydroxyisobutyrylated proteins identified in RH strain *T. gondii*, respectively. Overlap regions represent the number of proteins with both modifications in the two parasite strains. Ellipses in blue and yellow represent the numbers of crotonylated proteins and 2-hydroxyisobutyrylated proteins identified in ME49 strain *T. gondii*, respectively. *B*, Visual presentation of lysine crotonylation and 2-hydroxyisobutyrylation based on subcellular location in the two *T. gondii* strains. The outermost circle represents the number of proteins identified. The proteins associated with subcellular location are displayed by heat maps (black frame). The circle (navy blue) represents the number of modification sites. The relative abundance of each protein within a given organelle is represented by the red histograms. Circles in light blue and orange represent the number of 2-hydroxyisobutyrylation sites and crotonylation sites, respectively. A deeper color represents higher enrichment of the modification. Note that the height of each bar reflects the subcellular organelle abundance of each protein or PTM site, *i.e.* longer bars represent a greater degree of abundance. *C*, KEGG pathway-based enrichment analysis of the crotonylated and 2-hydroxyisobutyrylated proteins in the *T. gondii* strains (*p* < 0.05). Detailed data are listed in supplemental Data S8.

Modified peptides of both parasite strains were enriched with pan-antibodies specifically recognizing lysine residues with either crotonylation or 2-hydroxyisobutyrylation in three replicated experiments. Generally, the modification level of lysine 2-hydroxyisobutyrylation was significantly higher than that of crotonylation in *T. gondii,* irrespective of phenotype. In total, 1,061 and 984 proteins with 3,735 and 3,396 modified sites in RH and ME49 strain *T. gondii,* respectively, were crotonylated; 1,950 and 1,720 proteins with 9,502 and 8,092 sites in the two parasite strains were 2-hydroxyisobutyrylated, respectively (Fig. 1*B*, 3*A* and supplemental Data S5, S6). The number of proteins with mono-modification, either with crotonylation or 2-hydroxyisobutyrylation, was much less than that of proteins with multiple modifications. Further, 851 proteins (2,481 sites), accounting for 39.8% of all identified proteins, were modified by both crotonylation and 2-hydroxyisobutyrylation in the two parasite strains (supplemental Fig. S2).

Gene Ontology (GO) analyses indicated that lysine crotonylation was significantly enriched in the categories of cytoplasm, macromolecular complexes, which were predominantly related to protein translation, compound metabolism and biosynthesis processes in the two *T. gondii* strains (supplemental Data S7). However, the 2-hydroxyisobutyrylated proteins were mainly distributed in the nucleus, cytoplasm, and mitochondria in the two *T. gondii* strains, and the modified proteins participated in compound transport and metabolism processes. However, variation in distribution of the proteins with different modifications was also observed. As shown in Fig. 3*B*, lysine 2-hydroxyisobutyrylation and crotonylation were distributed in multiple cellular components of *T. gondii*, and the distribution of 2-hydroxyisobutyrylated proteins was much wider across the cytoplasm, whereas the crotonylated proteins were more concentrated in and around the nucleus. Functional characterization of these modified proteins suggested that they commonly regulate the pathways involved in ribosome, carbon metabolism, glycolysis/gluconeogenesis, proteasome and citrate cycle (tricarboxylic acid (TCA) cycle) processes in the two *T. gondii* strains (Fig. 3*C*).

##### Sequence Preference of Crotonylated and 2-Hydroxyisobutyrylated Peptides

The sequence context of the crotonylated and 2-hydroxyisobutyrylated lysines in the proteins was analyzed using the Motif-x program. Conserved crotonylation and 2-hydroxyisobutyrylation motifs were identified according to the criteria of specific amino acid sequences from ten amino acids upstream and downstream of the modified lysines (supplemental Fig. S3*A*–S3*D*). Isoleucine (I) and lysine (K) occurred most frequently upstream of the crotonylation sites, whereas leucine (L), lysine (K) and phenylalanine (F) were found most frequently downstream of the crotonylation sites. In addition, leucine (L), lysine (K), tyrosine (Y) and valine (V) occurred most frequently upstream of the 2-hydroxyisobutyrylation sites.

##### Quantitative Analysis of Crotonylated and 2-Hydroxyisobutyrylated Proteins

From further label-free quantitative analysis of modified proteins in the two parasite strains (RH/ME49), a total of 2,598 sites on 744 crotonylated proteins were quantifiable (supplemental Fig. S4 and supplemental Data S9). Of these proteins, 43 crotonylated proteins containing 52 upregulated sites and 40 crotonylated proteins containing 114 specific sites were detected in the *T. gondii* RH strain. A total of 106 crotonylated proteins containing 129 upregulated sites and 11 crotonylated proteins containing 46 specific sites were identified in the *T. gondii* ME49 strain. Additionally, we found that 6,753 2-hydroxyisobutyrylation sites on 1,349 proteins had quantitative information (supplemental Fig. S5 and supplemental Data S10). In total, 317 2-hydroxyisobutyrylated proteins containing 477 upregulated sites and 483 2-hydroxyisobutyrylated proteins containing 584 specific sites were found in the *T. gondii* RH strain. Moreover, 174 2-hydroxyisobutyrylated proteins containing 211 upregulated sites and 113 2-hydroxyisobutyrylated proteins containing 123 specific sites were detected in the *T. gondii* ME49 strain.

**Fig. 4.**
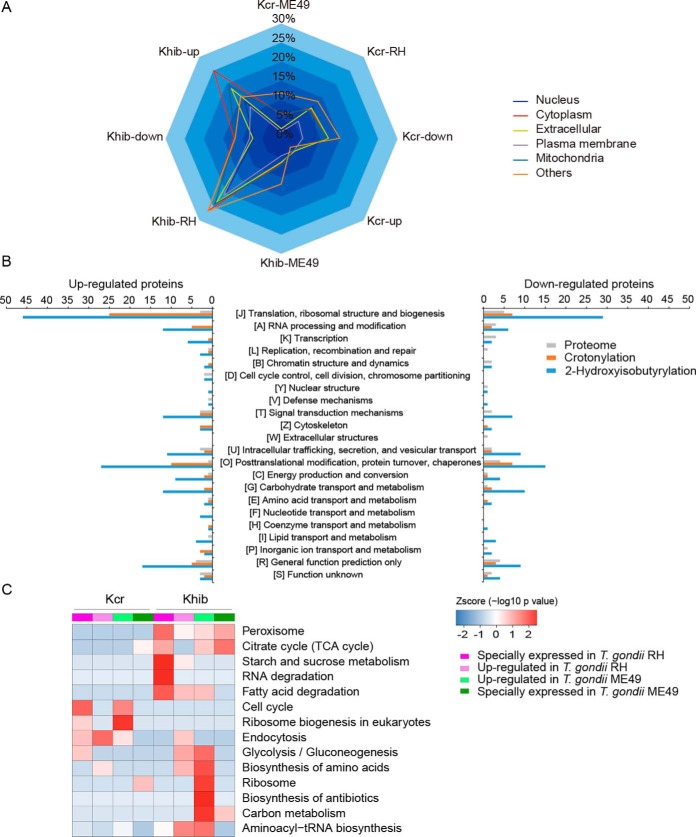
**Quantitative analysis of crotonylation and 2-hydroxyisobutyrylation in the phenotypically different *T. gondii* parasites.** *A*, The differentially crotonylated and 2-hydroxyisobutyrylated proteins were classified based on subcellular location in *T. gondii*. Detailed data are listed in supplemental Data S13. *B*, The differentially crotonylated and 2-hydroxyisobutyrylated proteins in the phenotypically different *T. gondii* strains were further analyzed by the KOG (Eukaryotic Orthologous Groups) database. The abscissa is the content of each category of KOG, and the ordinate is the number of proteins (Fisher's exact test, *p* < 0.05). Detailed data are listed in supplemental Data S14. *C*, KEGG pathway-based enrichment analysis at the differential modification level of crotonylated and 2-hydroxyisobutyrylated proteins in *T. gondii* (Fisher's exact test, *p* < 0.05). Detailed data are listed in supplemental Data S16 and S17.

Subcellular localization analysis revealed a wide distribution of proteins for differentially crotonylated and 2-hydroxyisobutyrylated proteins in the *T. gondii* RH strain. In the *T. gondii* ME49 strain, the differentially modified proteins were rarely located in the plasma membrane (Fig. 4*A*). Overall Eukaryotic Orthologous Group (KOG) analysis indicated that the differentially modified proteins were functionally clustered in the categories of translation, ribosomal structure and biogenesis, posttranslational modification, protein turnover, and chaperones in the two *T. gondii* strains (Fig. 4*B*).

To better investigate the biological function of the differentially modified proteins, we performed comparative analysis based on GO enrichment and Kyoto Encyclopedia of Genes and Genome (KEGG) pathway enrichment (Fig. 4*C* and supplemental Fig. S6–S8). Differentially lysine crotonylated and 2-hydroxyisobutyrylated proteins were predominantly enriched in the cytoplasm and ribosome, which may be involved in the regulation of tRNA aminoacylation, translation, compound metabolism and biosynthetic processes in *T. gondii*. Remarkably, differential crotonylation was enriched in endocytosis and the cell cycle in the *T. gondii* RH strain. The upregulated crotonylated proteins were related to tight junctions in the *T. gondii* ME49 strain. Notably, specific 2-hydroxyisobutyrylation was enriched in the myosin complex and actin cytoskeleton in the *T. gondii* RH strain, which may be essential for the regulation of motor function or rearrangement of the cytoskeleton. In addition, specific 2-hydroxyisobutyrylation was involved in fatty acid degradation and peroxisome in the *T. gondii* RH strain. Further, the upregulated 2-hydroxyisobutyrylation sites were highly enriched in aminoacyl-tRNA biosynthesis and glycolysis/gluconeogenesis in the *T. gondii* RH strain. In contrast, in the *T. gondii* ME49 strain, upregulated 2-hydroxyisobutyrylation was involved in aminoacyl-tRNA biosynthesis, amino acid biosynthesis and glycolysis/gluconeogenesis. Specific 2-hydroxyisobutyrylation was enriched in carbon fixation pathways in the *T. gondii* ME49 strain.

##### Crotonylation and 2-Hydroxyisobutyrylation of Histones

**Fig. 5.**
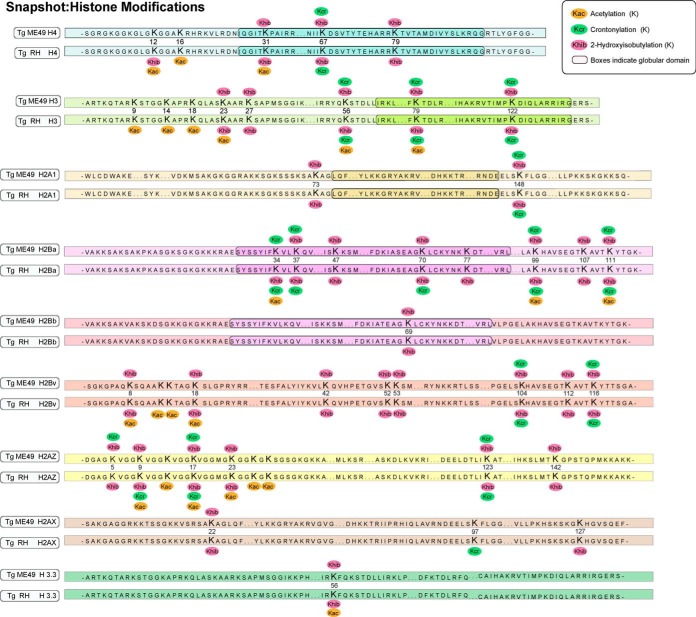
**Overview of lysine crotonylation and 2-hydroxyisobutyrylation sites identified on histones of the two *T. gondii* strains.** Green ellipses represent the lysine crotonylation sites. Purple ellipses represent the lysine 2-hydroxyisobutyrylation sites. Orange ellipses represent the lysine acetylation sites. Numbers in the middle of the sequences indicate the amino acid position on histon.

Histone modifications play a critical role in the epigenetic control of gene activation and silencing. A fine map of the canonical histones and histone variants with crotonylation and 2-hydroxyisobutyrylation was obtained. In total, 17 crotonylation sites and 37 2-hydroxyisobutyrylation sites were identified in *T. gondii* (Fig. 5). Among these sites, 16 crotonylation sites and 37 2-hydroxyisobutyrylation sites were detected on nine histones in the *T. gondii* RH strain. In addition, 14 crotonylation sites and 33 2-hydroxyisobutyrylation sites were identified in the *T. gondii* ME49 strain. In the *T. gondii* RH strain, the lysine residues H2AxK98, H2AzK10, and H2BaK71 were crotonylated, and H4K80, H2BaK35, H2BaK78, and H2AXK128 were 2-hydroxyisobutyrylated, whereas they were not modified in the *T. gondii* ME49 strain. Further, H2AzK6 was crotonylated in only the *T. gondii* ME49 strain. Post-translational modification of lysine residues on histones by acyl groups are important epigenetic markers with various biological functions. We also summarized the *T. gondii* histone acetylation sites (21) found on classical and variant histones in Fig. 5. Obviously, the three modifications were mainly abundant in H3, H4, H2Ba, H2Bv, H2Az. We discovered multiple modifications (Kcr, Khib, and Kac) on distinct lysine sites. Among them, three modifications were detected in H3K58, H3K80, H2BaK100, H2BaK112, H2AzK10, and H2AzK18, which play an important role in the epigenetic regulation of *T. gondii*. Acetylation sites associated with transcriptional activation included H3K9ac, H3K14ac, H3K18ac, H3K23ac, H4K12ac, and H4K16ac (22). It is noteworthy that only two 2-hydroxyisobutylation sites H3K23, H4K12 overlapped with them and no overlapping crotonylation sites were observed.

##### Crotonylation and 2-Hydroxyisobutyrylation of Transcriptional Regulators, the Spliceosomal Complex and Chromatin Remodeling Complexes

AP2 factors are considered the major transcription factors involved in both activation and repression of bradyzoite gene expression in *T. gondii* (23, 24). In this study, multiple crotonylation and 2-hydroxyisobutyrylation sites were detected on the AP2 factors. And numerous differential crotonylation and 2-hydroxyisobutyrylation modifications in histone modification enzymes were detected, including the arginine methyltransferase family (PRMT), histone deacetylase (HDAC2) and histone lysine acetyltransferase MYST-A (supplemental Table S1). Further, members of the RNA polymerase family (RBP1, RBP5, RBP9, and RBP11A), which plays an important role in gene transcription, were found to be modified by both crotonylation and 2-hydroxyisobutyrylation.

Pre-mRNA processing splicing factor has long been considered the “master regulator” of the spliceosome, the molecular machine that executes pre-mRNA splicing (25). Specific 2-hydroxyisobutyrylation of three sites (K526, K644, and K1055) and upregulated 2-hydroxyisobutyrylation of two sites (K566, K1508) on PRP8 were identified in the *T. gondii* RH strain. In contrast, in the *T. gondii* ME49 strain, 2-hydroxyisobutyrylation of two sites (K757 and K2315) on PRP8 was upregulated. Elongation factors (EFs) are protein factors that promote the elongation of polypeptide chains during mRNA translation. In this study, specific 2-hydroxyisobutyrylation of four sites on transcriptional EF (FACT80 K104; FACT140 K226, and K375; and SPT6 K2360) was detected in the *T. gondii* RH strain (supplemental Table S1).

##### Lysine Crotonylation and 2-Hydroxyisobutyrylation of Invasion-associated Proteins

**Fig. 6.**
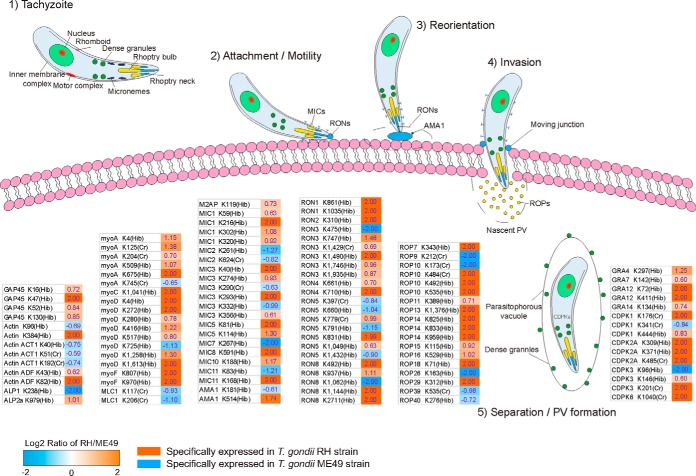
**Lysine crotonylation and 2-hydroxyisobutyrylation of invasion-related proteins.** Differential modification of key sites between the *T. gondii* RH and ME49 strains during invasion of host cells. 0 < Log2 of the RH/ME49 ratio < 2 represents upregulated modification of sites in the *T. gondii* RH strain. Log2 = 2 of the RH/ME49 ratio represents specific modification of sites in the *T. gondii* RH strain. −2 < Log2 of the RH/ME49 ratio < 0 represents upregulated modification of sites in the *T. gondii* ME49 strain. Log2 = −2 of the RH/ME49 ratio represents specific modification of sites in the *T. gondii* ME49 strain. Detailed data are listed in supplemental Data S19.

In this study, numerous proteins associated with parasite movement and gliding and moving junction (MJ) formation, proteins released from invasion-associated organelles and proteins associated with parasite egress after maturation were systematically analyzed for crotonylation and 2-hydroxyisobutyrylation. In total, 22 crotonylated proteins (32 sites) and 59 2-hydroxyisobutyrylated proteins (119 sites) showed differential modification levels between the two *T. gondii* strains (Fig. 6).

The machinery that powers tachyzoite gliding motility and penetration into host cells depends on parasite actin, the MyoA motor complex, and microneme component proteins (26). The motility of *T. gondii* is powered by the interaction of glideosome-associated proteins (GAPs), actin-related proteins (actin ACT1), actin depolymerizing factor (ADF), actin-like proteins (ALP1 and ALP2a), myosin light chain (MLC1) and several myosins. In general, modification of invasion-associated proteins was more prominent in the RH strain than in the ME49 strain. Crotonylation of two sites (K125 and K204) on myosin A was upregulated in the *T. gondii* RH strain. However, upregulated crotonylation of lysine residues (actin ACT1 K51 and K192; MLC1 K117 and K206) was also detected in the *T. gondii* ME49 strain. Further, specific 2-hydroxyisobutyrylation of GAP45 (K47), actin (K384), ADF (K82), myosin A (K675), myosin C (K1041), myosin D (K4, K272, and K1613), and myosin F (K807 and K970) and upregulated 2-hydroxyisobutyrylation of residues in GAP45 (K16, K52, and K130), ADF (K43), ALP2a (K979), myosin A (K4 and K509), and myosin D (K280, K416, K517, and K1258) were detected in the *T. gondii* RH strain. Further, specific 2-hydroxyisobutyrylation of a site on ALP1 (K238) and upregulated 2-hydroxyisobutyrylation of actin (K96), ACT1 (K40), myosin A (K745) and myosin D (K725) were detected in the *T. gondii* ME49 strain. Not only specific crotonylation of sites (K401 and K1138) and specific 2-hydroxyisobutyrylation of lysine K354 of the membrane skeleton protein IMC2A were detected in the *T. gondii* RH strain, other microtubule-associated proteins, such as SPM1, beta-tubulin and alpha-tubulin TUBA1, which are important for parasite cytoskeleton structure, were also modified in the two *T. gondii* strains.

Further, our global analysis revealed novel crotonylated and 2-hydroxyisobutyrylated proteins in micronemes and rhoptries. The proteins secreted from these organelles are involved in the cellular signaling process of *T. gondii* invasion into the host. Upregulated crotonylation of lysines (MIC2 K624, and MIC3 K290) was detected in the *T. gondii* ME49 strain. Specific 2-hydroxyisobutyrylation of MIC1 (K216), MIC3 (K40 and K293), MIC5 (K81), MIC8 (K591) and MIC11 (K168) and upregulation of 2-hydroxyisobutyrylation of MIC1 (K59, K302, and K320), MIC3 (K274 and K356), MIC5 (K114), and MIC10 (K188) were identified in the *T. gondii* RH strain. In contrast, specific 2-hydroxyisobutyrylation of MIC7 (K267) and upregulation of 2-hydroxyisobutyrylation of MIC2 (K261), MIC3 (K332), and MIC11 (K83) were found in the *T. gondii* ME49 strain. Notably, subcellular localization indicated that all modified MICs were in extracellular except that MIC8 was located in the peroxisome.

**Fig. 7.**
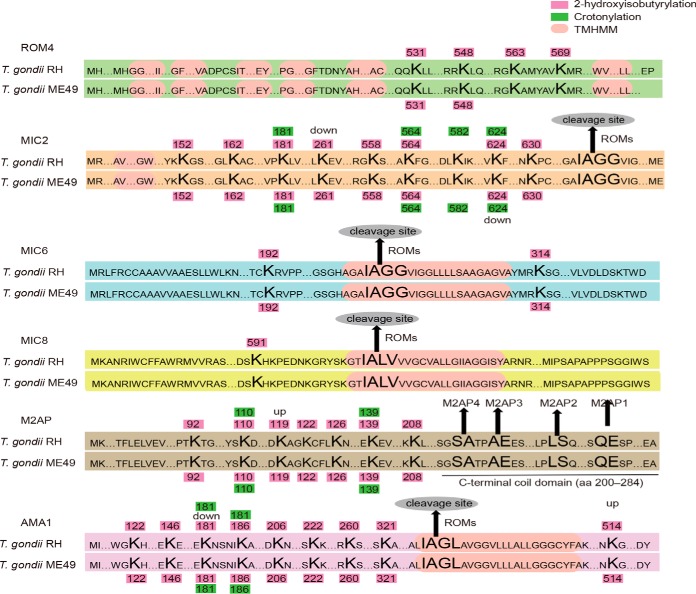
**Overview of lysine crotonylation and 2-hydroxyisobutyrylation sites identified on micronemes of the two *T. gondii* strains.** Green ellipses represent lysine crotonylation sites. Purple ellipses represent lysine 2-hydroxyisobutyrylation sites. C-terminal cleavage sites for AMA1, M2AP, MIC2, MIC6 and MIC8 are marked with arrows.

During invasion, the parasite forms a specific structure called a MJ with the host-cell surface, which is mediated by parasite proteins secreted from the microneme and rhoptry organelles (27). Differential crotonylation and 2-hydroxyisobutyrylation of rhoptry-related proteins in the parasite strains were observed. The apical membrane antigen-1 (AMA1-RON2) complex bridges the parasite and host cell and plays a key role in invasion (28). Upregulation of 2-hydroxyisobutyrylation of K514 of AMA1 and down-regulation of 2-hydroxyisobutyrylation of K181 of AMA1 were detected in the *T. gondii* RH strain compared with the ME49 strain. Specific 2-hydroxyisobutyrylation of RON2 (K310) was detected in the *T. gondii* RH strain. Rhomboid protease TgROM4 plays a vital role in parasite replication by cleaving TgAMA1 to commence replication, followed by invasion (29). Two lysine residues (K563 and K569) of ROM4 were 2-hydroxyisobutyrylated in only the *T. gondii* RH strain (Fig. 7). In addition, modification was widely distributed in the proteins of the RON family, especially of the RH strain. Crotonylation of K1429 of RON3 and RON5 K779 were upregulated in the *T. gondii* RH strain in contrast to upregulation of crotonylation of RON5 K397 in the *T. gondii* ME49 strain. Moreover, 2-hydroxyisobutyrylation of RON3 (K747, K661, K1746, and K1935), RON5 (K831 and K1049), and RON8 K937 was upregulated, and 2-hydroxyisobutyrylation of K861 and K1035 of RON1; K1490 of RON3; K710 of RON4; and K492, K1144, and K2711 of RON8 was detected in only the *T. gondii* RH strain. In the *T. gondii* ME49 strain, upregulated 2-hydroxyisobutyrylation occurred at only two sites (RON5 K660, K791 and K1432), and specific 2-hydroxyisobutyrylation occurred at RON3 K475 and RON8 K1062.

Likewise, the ROP protein family was highly modified in *T. gondii*. Crotonylation of ROP10 K484 and 2-hydroxyisobutyrylation of eight residues (ROP7 K343; ROP10 K492, and K535; ROP13 K1376; ROP14 K825, K833, and K959; ROP18 K71; and ROP29 K312) occurred in only the RH strain. Additionally, 2-hydroxyisobutyrylation of ROP7 K315, ROP11 K389, ROP15 K115, and ROP16 K529 was upregulated in the RH strain. However, specific 2-hydroxyisobutyrylation of ROP26 K163 and crotonylation of two residues (ROP9 K212 and ROP10 K173) was detected in only the ME49 strain. Additionally, specific 2-hydroxyisobutyrylation of GRA12 (K72 and K411) and upregulated 2-hydroxyisobutyrylation of GRA4 K297, GRA7 K142, and GRA14 K134 occurred in the *T. gondii* RH strain.

Calcium-dependent protein kinases (CDPKs) contribute to several aspects of the life cycle of *T. gondii,* such as gliding motility, cell invasion, and egress, as well as some other critical developmental processes (30). Prevalent modifications in the CDPK family were also observed in the RH strain. Specific crotonylation of four residues (CDPK1 K176, CDPK2A K485, CDPK3 K201, and CDPK6 K1040) and 2-hydroxyisobutyrylation of two sites (CDPK2A K309 and K371) were observed in the RH strain, and upregulation of 2-hydroxyisobutyrylation of two sites (CDPK1 K444 and CDPK3 K146) was identified in the *T. gondii* RH strain. Additionally, specific 2-hydroxyisobutyrylation of CDPK3 K96 and upregulated crotonylation of CDPK1 K341 were detected in *T. gondii* ME49 strain.

##### Modification of Enzymes Involved in Glycolysis/Gluconeogenesis and the Citrate Cycle (TCA Cycle)

Lysine crotonylation and 2-hydroxyisobutyrylation of the key enzymatic components mediating glycolysis/gluconeogenesis, citrate cycle (TCA cycle) and peroxisome metabolism pathways were deeply analyzed in *T. gondii* (supplemental Fig. S9*A*, supplemental Data S20). In total, 17 enzymes involved in glycolysis/gluconeogenesis were differentially modified (supplemental Table S2). The most significantly modified protein was the rate-limiting enzyme phosphofructokinase PFKII; 2-hydroxyisobutyrylation of lysines K250 and K268 located in the active domain of this enzyme was upregulated, which may have important regulatory effects on substrate binding and the glucose metabolism pathway (supplemental Fig. S9*B* and S9*C*).

For the citrate cycle, differentially crotonylated and 2-hydroxyisobutyrylated sites were observed in seven enzymes, including the key regulatory enzymes citrate synthase and isocitrate dehydrogenase (supplemental Table S3). Glutamine also confers a much-needed pool of nitrogen for nucleotide and protein syntheses, and extracellular parasites can use either of the two nutrients to produce enough energy for host-cell invasion (31). Several differential modification sites on multiple glutamine-related proteins were identified (supplemental Table S3), which have an important influence on energy regulation. Further, catalase (CAT) is a marker enzyme of peroxisomes and a potent H_2_O_2_-detoxifying enzyme in *T. gondii* (32). In this study, specific 2-hydroxyisobutyrylation of CAT K240 of the *T. gondii* RH strain and specific 2-hydroxyisobutyrylation of CAT K222 of the ME49 strain were detected.

##### Crotonylated and 2-Hydroxyisobutyrylated Proteins Involved in Protein Biosynthesis, Folding, and Ubiquitin-dependent Degradation

Seven proteins with differential crotonylation and fifteen proteins with differential 2-hydroxyisobutyrylation were observed in relation to the aminoacyl-tRNA biosynthesis pathway, which may be involved in the regulation of protein biosynthesis in the two *T. gondii* strains (supplemental Fig. S10 and supplemental Table S4). Meanwhile, several differentially modified heat shock proteins (HSP20, HSP28, HSP29, HSP60, HSP70, and HSP90) were detected, which may assist in protein folding to avoid abnormal folding and aggregation (supplemental Table S5). In addition, several ubiquitin-proteasome-related proteins (ubiquitin-activating enzyme E1, ubiquitin C-terminal hydrolase UCHL3 and proteasome 26S regulatory subunit) were crotonylated and 2-hydroxyisobutyrylated in the phenotypically different *T. gondii* parasites (supplemental Data S21).

##### The Interaction Network of Differentially or Uniquely Crotonylated and 2-Hydroxyisobutyrylated Proteins

In total, 195 differentially modified proteins were mapped to a protein interaction database, presenting a global view of the interaction network (supplemental Fig. S11). The proteins were related to mainly the biosynthesis of ribosomes, aminoacyl-tRNA biosynthesis, glycolysis/gluconeogenesis and TCA cycle in the two phenotypically different *T. gondii* strains. The significant hub proteins included actin-like protein ALP2a, UV excision repair protein Rad23, threonyl-tRNA synthetase family protein, peptidyl-prolyl cis-trans isomerase, actin ACT1, T-complex protein 1 delta subunit, snoRNA-binding domain-containing protein and pyruvate dehydrogenase complex subunit PDH-E3II, which had a high degree of network interaction.

We also established interaction networks of 207 specifically modified proteins via STRING and Cytoscape software. As a result, these proteins were significantly related to not only the ribosome, aminoacyl-tRNA biosynthesis, TCA cycle and glycolysis/gluconeogenesis but also the spliceosome and proteasome. As shown in supplemental Fig. S12, the proteins had a high degree of network interaction, such as heat shock protein Hsp70, UV excision repair protein Rad23, carbamoylphosphate synthetase, peptidyl-prolyl cis-trans isomerase, acetyl-CoA carboxylase ACC1, actin-like protein ALP1, RNA recognition motif-containing protein, Ras-related protein Rab11, and DnaJ family Sec63 protein, comprising a dense protein interaction network. Collectively, our findings suggested that these proteins were relatively active in a diverse array of biological processes, although lysine crotonylation occurred at a low level.

## DISCUSSION

Protein PTMs play fundamental roles in the regulation of most biological events. PTMs are primarily enzymatic modifications of proteins that occur after protein translation, which alter protein physical or chemical properties, cellular location, functionality or stability (8). Common PTMs include succinylation, phosphorylation, methylation, acetylation, glycosylation, and ubiquitination (33).

Apicomplexan parasites such as *T. gondii* undergo a complex two-host life cycle involving multiple developmental stages and nucleated cells. One of the mechanisms by which parasites likely respond to extracellular stimuli and life cycle transitions is through PTMs (8). PTMs can build a favorable condition for the parasite, including motility, invasion, replication and egress (8).

Lysine crotonylation and 2-hydroxyisobutyrylation are two new types of protein acylation PTMs. As shown in Table I, studies on the two PTMs have been extended from histone to non-histone modifications in a variety of organisms, from human being to plants (34–47). Histone crotonylation is a new type of lysine acylation PTM that is enriched at active gene promoters and potential enhancers in yeast and mammalian cells (34). Previous research suggested that histone lysine 2-hydroxyisobutyrylation is involved in male cell differentiation and plays a critical role in the regulation of chromatin functions in animals (38). However, information regarding protein lysine crotonylation and 2-hydroxyisobutyrylation in *T. gondii* is still limited. In this study, we conducted a global analysis of lysine crotonylation and 2-hydroxyisobutyrylation in two phenotypically different *T. gondii* strains, the RH strain and ME49 strain. RH strain *T. gondii* is a parasite with fast proliferation capacity and high virulence in murine model animals, but the ME49 strain grows much slower and is less virulent than the RH strain (48). Here, an atlas of lysine crotonylation and 2-hydroxyisobutyrylation of the proteomes of the two parasite stains was established. Qualitative analysis indicated that 1061 and 984 proteins with 3735 and 3396 modified sites in RH and ME49 strain *T. gondii*, respectively, were crotonylated, and 1950 and 1720 proteins with 9502 and 8092 2-hydroxyisobutyrylation sites, respectively, in the two parasite strains were identified. In addition to the finding that modification of proteins was more prevalent in the RH strain than in the ME49 strain, lysine crotonylation and 2-hydroxyisobutyrylation were the most prominent PTMs in *T. gondii* except for phosphorylation (11, 12, 49–57) (phosphorylation can occur on diverse amino acids) (supplemental Table S6), suggesting critical roles of crotonylation and 2-hydroxyisobutyrylation in the regulation of various biological processes of the parasites.

**Table I:** Lysine crotonylation and 2-hydroxyisobutyrylation in different species

Types	Species	Proteins	Sites	Function	Reference
Kcr	Carica papaya L.	2,120	5,995	Biosynthesis of antibiotics, carbon metabolism, biosynthesis of amino acids, glycolysis, microbial metabolism	47
	Nicotiana tabacum	637	2,044	Carbon metabolism, citrate cycle, glycolysis, biosynthesis of amino acids, photosynthesis, biosynthesis, folding, and degradation of proteins	37
	Zebrafish embryos	218	557	Muscle contraction and protein synthesis	45
	Tea plants	971	2,288	Photosynthesis, carbon fixation and amino acid metabolism	46
	Human lung adenocarcinoma cell line H1299	1,024	2,696	Ribosome, spliceosome, proteasome and Parkinson's disease pathways	35
	*Rhodotorula mucilaginosa*	629	1,691	Chromatin dynamics, gene expression, and metabolic pathways	44
	*Oryza sativa* L. *japonica* (rice)	690	1,265	Photosynthesis, ribosome, oxidative phosphorylation, and proteasome	42
	Human somatic and mouse male germ cell		67	Active promoters and potential enhancers, active sex chromosome	34
	*T. gondii* RH	1,061	3,735	Ribosome, proteasome, pentose phosphate pathway, microbial metabolism in diverse environments, aminoacyl−tRNA biosynthesis, glycolysis/ gluconeogenesis, citrate cycle (TCA cycle), carbon metabolism, biosynthesis of amino acids, peroxisome, carbon fixation in photosynthetic organisms, 2-oxocarboxylic acid metabolism, glutathione metabolism, MAPK signaling pathway, ferroptosis, gap junction	
	*T. gondii* ME49	984	3,396	Ribosome, proteasome, pentose phosphate pathway, microbial metabolism in diverse environments, citrate cycle (TCA cycle), glycolysis/gluconeogenesis, aminoacyl−tRNA biosynthesis, carbon metabolism, RNA transport, oxidative phosphorylation	
Khib	Male germ cells		63	Male cell, differentiation, chromatin functions	38
	*Physcomitrella patens*	3,001	11,976	Microbial metabolism in diverse environments, carbon metabolism, biosynthesis of antibiotics	43
	Rice Seeds (*Oryza sativa*)	2,512	9,916	Glycolysis/gluconeogenesis, TCA cycle, starch biosynthesis, lipid metabolism, protein biosynthesis and processing	39
	*Proteus mirabilis*	1,051	4,735	Purine metabolism, pentose phosphate pathway and glycolysis/gluconeogenesis	41
	*T. gondii* RH	1,950	9,502	Ribosome, proteasome, glycolysis/gluconeogenesis, citrate cycle (TCA cycle), carbon metabolism, biosynthesis of amino acids, carbon fixation in photosynthetic organisms, spliceosome, peroxisome	
	*T. gondii* ME49	1,720	8,092	Ribosome, proteasome, glycolysis/gluconeogenesis, citrate cycle (TCA cycle), carbon metabolism, aminoacyl−tRNA biosynthesis, carbon fixation in photosynthetic organisms, alanine, aspartate and glutamate metabolism	

Crotonylation (Kcr); 2-hydroxyisobutyrylation (Khib).

Histone modifications play an essential role in epigenetic control of gene activation and silencing (24). Histone subunits H2A and H2B are not as conserved as H3 and H4 (43), and the histone 2-hydroxyisobutyrylation pattern in *T. gondii* was complex. In this study, unlike lysine crotonylation (occurring mainly at the N termini of the histones), lysine 2-hydroxyisobutyrylation is not only located in N-terminal tail domains but also abundant in other regions of histones. For example, five 2-hydroxyisobutyrylation sites were located in the H2B globular domain between K35 and K78. Further, we observed five 2-hydroxyisobutyrylation sites (H3K57, H3K80, H3K123, H4K32, and H4K80) that are also conserved in humans, mouse cells, *Physcomitrella patens* and rice seeds (34, 39, 43). As shown in Fig. 5 and supplemental Table S7, a map of lysine crotonylation and 2-hydroxyisobutyrylation was established for *T. gondii* histones, with patterns like previously reported histone modifications (11, 22, 49, 54). However, lysine 2-hydroxyisobutyrylation is a more abundant modification than other histone-modified markers. Though most lysine residues were mono-modified, multiple modifications on H3K24, H4K32, H3K57, H2AzK10, H2AzK18, H2BaK100 and H2BaK112 were observed, indicating that they are critical epigenetic markers in the development of the parasites. A previous study indicated a tight association between H4K8hib and transcriptional activity (38). However, we did not detect that modification in *T. gondii*. Histone crotonylation is as dynamic as histone acetylation and is not functionally redundant with acetylation but is critically important for global transcription in mammalian cells (58). Therefore, despite the low abundance of histone crotonylation, it may play an important role in the transcription process of *T. gondii*. Earlier studies defined the evolutionarily conserved YEATS domain as a family of crotonyllysine readers and specifically demonstrated that the YEATS domain of AF9 directly linked histone crotonylation to active transcription (59). In *T. gondii*, TGME49_215730 (Gas41, putative) also contains a YEATS domain, indicating that it may also be a crotonyllysine reader associated with active transcription.

The modified proteins distributed in different cell compartments were detected, and they likely contributed to a wide range of functions, such as glycolysis/gluconeogenesis, TCA cycle, ribosome, biosynthesis of amino acids, carbon metabolism and proteasome processes. Moreover, the crotonylated proteins of the *T. gondii* RH strain were enriched in previously unreported pathways such as peroxisome, glutathione metabolism, MAPK signaling pathway, ferroptosis and gap junction (Fig. 3*C*), which indicated that crotonylation may regulate these pathways to allow the parasite to adapt to external stimuli for its own development and reproduction. Further, 2-hydroxyisobutyrylation was enriched in the spliceosome of the RH strain and played an important role in regulating genetic activity (Fig. 3*C*). When mitochondria cannot fuse and elongate or cannot oxidize fatty acids, parasite growth is enhanced (60). Here, specifically 2-hydroxyisobutyrylated proteins in the *T. gondii* RH strain were mostly related to fatty acid degradation (Fig. 5*C*), which suggests that this modification facilitates fatty acid oxidation to fulfil the energy generation requirements of the rapid proliferation of the parasite. In addition, we found that multiple differential or specifically modified rate-limiting enzymes were involved in a series of energy processes, such as hexokinase, 6-phosphofructokinase, pyruvate kinase and citrate synthase I, and most of the upregulated and specific modifications were found in the *T. gondii* RH strain.

Host-cell recognition and invasion are essential steps for parasite proliferation in mammalian hosts. To be able to invade a host cell, tachyzoites must attach to the host-cell surface through interaction of parasite surface proteins (MICs) with host-cell receptors such as sialic acid and heparin, and AMA1 subsequently forms a MJ with the rhoptry neck RON2 protein for a firm anchorage to their host cell (61). In this process, parasites have a unique machinery called the glideosome, which is composed of an actomyosin system that underlies the plasma membrane (62). The glideosome promotes substrate-dependent gliding motility, which powers migration across biological barriers as well as active host-cell entry and egress from infected cells (62). We found that lysine crotonylation and 2-hydroxyisobutyrylation were widely distributed in proteins associated with cell invasion. Here, a total of 70 specific modification sites on invasion-related proteins, such as gliding-associated proteins, rhoptries, micronemes and dense particles, were identified in the two *T. gondii* strains, and 61 (87.14%) sites were detected in the RH strain (Fig. 6, supplemental Data S19). Of these 61 sites, 52 (85.25%) were 2-hydroxyisobutyrylated in the RH strain, indicating that 2-hydroxyisobutyrylation may promote the function of the proteins and contribute to rapid invasion of host cells. Given that these proteins are essential elements in the host-cell invasion process, lysine crotonylation and 2-hydroxyisobutyrylation may co-regulate protein release from specialized organelles, processing, and complex formation during the highly coordinated process of host-cell invasion. Further, these highly modified invasion-related proteins are distributed in mainly the apical complex, including three secretory organelles (microneme, rhoptry, and dense granule). Modified proteins such as AMA1, M2AP, and MIC2, which are differentially expressed, are produced by alternative splicing. Rhomboid proteases (ROMs) are thought to cleave adhesins within their transmembrane segments, thus allowing the parasite to disengage from receptors and completely enter the host cell (63). The cleavage site sequence (IAGG/IALV/IAGL), mapped in the *T. gondii* microneme proteins TgAMA1, TgM2AP, TgMIC2, TgMIC6, and TgMIC8, is conserved in microneme proteins of other apicomplexans (64–66). In our study, TgAMA1, TgM2AP, and TgMIC2 were highly modified, and all modification sites except for AMA1 K541 were located on the upstream side of the cleavage site (Fig. 7). Previous studies suggested that ROM4 was the primary protease involved in adhesin processing and host-cell invasion (63). We detected specific 2-hydroxyisobutyrylation of three sites, ROM4 K563 and K569 and MIC8 K591, in the *T. gondii* RH strain. The data indicated that ROM4 is highly modified, and thus, 2-hydroxyisobutyrylation could be a mechanism to organize enzymes and substrates, which may be involved in the regulation of ROM activity, promoting rapid host cell invasion.

ROP5, which is involved in invasion and virulence by increasing ROP18 kinase activity, was palmitoylated (51). Palmitoylation may regulate ROP5's ability to interact with ROP18 and other binding partners, some of which are determinants of *T. gondii* virulence in mice, and its affinity as a competitive inhibitor of immunity-related GTPase (IRG) oligomerization (67–69). In this study, ROP5 and ROP18 were modified. Although there was no difference in the modification of ROP5 between the two strains, a specific 2-hydroxyisobutyrylation of ROP18 K71 in the *T. gondii* RH strain was observed. Crotonylation and 2-hydroxyisobutyrylation may modulate the interaction between ROP5 and ROP18. However, the significance of specific modifications associated with parasite virulence still needs further experimental verification.

The transition between tachyzoite and bradyzoite is epigenetically regulated and coupled to the cell cycle (24). The most common mechanism of epigenetic gene regulation involves alteration of chromatin structure. Regulation of euchromatin or heterochromatin production by transcriptional complexes and polymerases and AP2 factors are considered the major transcription factors in *T. gondii* (24). In our study, the AP2 transcription factors AP2X-4 and AP2X-7 were modified, but no differential modifications were observed in the two strains. However, differential or specific lysine modifications were detected on RNA polymerases, such as RPB1 K179, RPB5 K945 and K958, RPB9 K279 and RPB11A K576 and K1,979 (supplemental Table S1). The arginine methyltransferase family (PRMT) has been implicated in a variety of cellular processes, including signal transduction, epigenetic regulation, and DNA repair pathways (70). The MYST family of lysine acetyltransferases (KATs) functions in a wide variety of cellular operations, including gene regulation and the DNA damage response (71). Histone deacetylases (HDACs) comprise a family of enzymes that participate in the regulation of chromatin structure, gene expression, and cell signaling in eukaryotes (72). Here, downregulated crotonylation (MYST-A K54), specific 2-hydroxyisobutyrylation (PRMT4 K1,689) and upregulated 2-hydroxyisobutyrylation (PRMT2 K935; HDAC2 K1,226) were observed in the *T. gondii* RH strain. Thus, lysine crotonylation and 2-hydroxyisobutyrylation of the AP2 transcription factors may also regulate the transition between tachyzoite and bradyzoite.

In conclusion, the global maps of lysine crotonylation and 2-hydroxyisobutyrylation of the two phenotypically different *T. gondii* strains will provide a valuable resource to facilitate the illumination of the biology of the zoonotic *T. gondii* parasite and to search for new tools for better disease control.

## DATA AVAILABILITY

The MS proteomics data have been deposited to the ProteomeXchange Consortium (http://proteomecentral.proteomexchange.org) via the PRIDE partner repository with the data set identifier PXD014133. All spectra files have been successfully uploaded to MS-Viewer (http://msviewer.ucsf.edu/prospector/cgi-bin/msform.cgi?form=msviewer). The unique search keys for different omics projects are list here. For qualitative identification: 2-hydroxyisobutyrylation- *T. gondii* ME49 strain (20gwwuog0n), 2-hydroxyisobutyrylation- *T. gondii* RH strain (xexcqiyxkv), Crotonylation- *T. gondii* ME49 strain (t0weiscwsh), Crotonylation- *T. gondii* RH strain (lknajnxrrm). For quantitative identification: Proteome (pjeuhbjqbv), 2-hydroxyisobutyrylation (sll57iaxfj), Crotonylation (faslt9iyhb).

## Supplementary Material

supplemental Data S1, S2

Supplementary figures

Supplementary dataset 1

Supplementary dataset 2

Supplementary dataset 3

Supplementary dataset 4

Supplementary dataset 5

Supplementary dataset 6

Supplementary dataset 7

Supplementary dataset 8

Supplementary dataset 9

Supplementary dataset 10

Supplementary dataset 11

Supplementary dataset 12

Supplementary dataset 13

Supplementary dataset 14

Supplementary dataset 15

Supplementary dataset 16

Supplementary dataset 17

Supplementary dataset 18

Supplementary dataset 19

Supplementary dataset 20

Supplementary dataset 21

Supplementary dataset 22

Supplementary dataset 23

## Footnotes

^1^ The abbreviations used are:

DC: dendritic cell
PTM: post-translational modification
IP: immunoprecipitation
NSI: nanospray ionization
IRG: immunity-related GTPase.

## References

[1] Marino N. D., Panas M. W., Franco M., Theisen T. C., Naor A., Rastogi S., Buchholz K. R., Lorenzi H. A., and Boothroyd J. C. (2018) Identification of a novel protein complex essential for effector translocation across the parasitophorous vacuole membrane of *Toxoplasma gondii*. PLoS Pathog. 14, e1006828 29357375 10.1371/journal.ppat.1006828 PMC5794187

[2] Kwong W. K., del Campo J., Mathur V., Vermeij M. J. A., and Keeling P. J. (2019) A widespread coral-infecting apicomplexan with chlorophyll biosynthesis genes. Nature 568, 103–107 30944491 10.1038/s41586-019-1072-z

[3] Osunkalu V. O., Akanmu S. A., Ofomah N. J., Onyiaorah I. V., Adediran A. A., Akinde R. O., and Onwuezobe I. A. (2011) Seroprevalence of *Toxoplasma gondii* IgG antibody in HIV-infected patients at the Lagos University Teaching Hospital. HIV AIDS 3, 101–105 10.2147/HIV.S15532 PMC3218715 22096412

[4] Pappas G., Roussos N., and Falagas M. E. (2009) Toxoplasmosis snapshots: global status of *Toxoplasma gondii* seroprevalence and implications for pregnancy and congenital toxoplasmosis. Int. J. Parasitol. 39, 1385–1394 19433092 10.1016/j.ijpara.2009.04.003

[5] Valentini P., Buonsenso D., Barone G., Serranti D., Calzedda R., Ceccarelli M., Speziale D., Ricci R., and Masini L. (2015) Spiramycin/cotrimoxazole versus pyrimethamine/sulfonamide and spiramycin alone for the treatment of toxoplasmosis in pregnancy. J. Perinatol. 35, 90–94 25211284 10.1038/jp.2014.161

[6] Weiss L. M., Fiser A., Angeletti R. H., and Kim K. (2009) Toxoplasma gondii proteomics. Expert Rev. Proteomics 6, 303–313 19489701 10.1586/epr.09.16 PMC2741161

[7] Witze E. S., Old W. M., Resing K. A., and Ahn N. G. (2007) Mapping protein post-translational modifications with mass spectrometry. Nat. Method 4, 798–806 10.1038/nmeth1100 17901869

[8] Yakubu R. R., Weiss L. M., and Silmon de Monerri N. C. (2018) Post-translational modifications as key regulators of apicomplexan biology: insights from proteome-wide studies. Mol. Microbiol. 107, 1–23 29052917 10.1111/mmi.13867 PMC5746028

[9] Croken M. M., Nardelli S. C., and Kim K. (2012) Chromatin modifications, epigenetics, and how protozoan parasites regulate their lives. Trends Parasitol. 28, 202–213 22480826 10.1016/j.pt.2012.02.009 PMC3340475

[10] Fauquenoy S., M. W., Hovasse A., Bednarczyk A., Slomianny C., Schaeffer C., Van Dorsselaer A., and Tomavo S. (2008) Proteomics and glycomics analyses of N-glycosylated structures involved in *Toxoplasma gondii*–host cell interactions. Mol. Cell Proteomes 7, 891–910 10.1074/mcp.M700391-MCP200 18187410

[11] Silmon de Monerri N. C., Yakubu R. R., Chen A. L., Bradley P. J., Nieves E., Weiss L. M., and Kim K. (2015) The ubiquitin proteome of *Toxoplasma gondii* reveals roles for protein ubiquitination in cell-cycle transitions. Cell Host Microbe 18, 621–633 26567513 10.1016/j.chom.2015.10.014 PMC4968887

[12] Braun L., Cannella D., Pinheiro A. M., Kieffer S., Belrhali H., Garin J., and Hakimi M. A. (2009) The small ubiquitin-like modifier (SUMO)-conjugating system of *Toxoplasma gondii*. Int. J. Parasitol. 39, 81–90 18761012 10.1016/j.ijpara.2008.07.009

[13] Gissot M., Kelly K. A., Ajioka J. W., Greally J. M., and Kim K. (2007) Epigenomic modifications predict active promoters and gene structure in *Toxoplasma gondii*. PLoS Pathog. 3, e77 17559302 10.1371/journal.ppat.0030077 PMC1891328

[14] Xiao H., El Bissati K., Verdier-Pinard P., Burd B., Zhang H., Kim K., Fiser A., Angeletti R. H., and Weiss L. M. (2010) Post-translational modifications to *Toxoplasma gondii* alpha- and beta-tubulins include novel C-terminal methylation. J. Proteome Res. 9, 359–372 19886702 10.1021/pr900699a PMC2813730

[15] Grigg M. E., Bonnefoy S., Hehl A. B., Suzuki Y., and Boothroyd J. C. (2001) Success and virulence in *Toxoplasma* as the result of sexual recombination between two distinct ancestries. Science 294, 161–165 11588262 10.1126/science.1061888

[16] Angeloni M. B., Guirelli P. M., Franco P. S., Barbosa B. F., Gomes A. O., Castro A. S., Silva N. M., Martins-Filho O. A., Mineo T. W., Silva D. A., Mineo J. R., and Ferro E. A. (2013) Differential apoptosis in BeWo cells after infection with highly (RH) or moderately (ME49) virulent strains of *Toxoplasma gondii* is related to the cytokine profile secreted, the death receptor Fas expression and phosphorylated ERK1/2 expression. Placenta 34, 973–982 24074900 10.1016/j.placenta.2013.09.005

[17] Wang W., Huang P., Jiang N., Lu H., Zhang D., Wang D., Zhang K., Wahlgren M., and Chen Q. (2018) A thioredoxin homologous protein of *Plasmodium falciparum* participates in erythrocyte invasion. Infect Immun. 86 10.1128/IAI.00289-18 PMC6056854 29844242

[18] Wang W., Liu F., Jiang N., Lu H., Yang N., Feng Y., Sang X., Cao Y., and Chen Q. (2018) *Plasmodium* TatD-like DNase antibodies blocked parasite development in the mosquito gut. Front. Microbiol. 9, 1023 29867907 10.3389/fmicb.2018.01023 PMC5968200

[19] Szklarczyk D., Franceschini A., Kuhn M., Simonovic M., Roth A., Minguez P., Doerks T., Stark M., Muller J., Bork P., Jensen L. J., and von Mering C. (2011) The STRING database in 2011: functional interaction networks of proteins, globally integrated and scored. Nucleic Acids Res. 39, D561–D568 21045058 10.1093/nar/gkq973 PMC3013807

[20] Doerks T., Copley R. R., Schultz J., Ponting C. P., and Bork P. (2002) Systematic identification of novel protein domain families associated with nuclear functions. Genome Res. 12, 47–56 11779830 10.1101/gr.203201 PMC155265

[21] Jeffers V., and Sullivan W. J. Jr. (2012) Lysine acetylation is widespread on proteins of diverse function and localization in the protozoan parasite *Toxoplasma gondii*. Eukaryotic cell 11, 735–742 22544907 10.1128/EC.00088-12 PMC3370464

[22] Nardelli S. C., Che F. Y., Silmon de Monerri N. C., Xiao H., Nieves E., Madrid-Aliste C., Angel S. O., Sullivan W. J. Jr, Angeletti R. H., Kim K., and Weiss L. M. (2013) The histone code of *Toxoplasma gondii* comprises conserved and unique posttranslational modifications. MBio 4, e00922–13 24327343 10.1128/mBio.00922-13 PMC3870261

[23] Hong D. P., Radke J. B., White M. W. (2017) Opposing transcriptional mechanisms regulate *Toxoplasma* development. mSphere 2, e00347–16 28251183 10.1128/mSphere.00347-16 PMC5322347

[24] K., K. (2018 6 22) The epigenome, cell cycle, and development in *Toxoplasma*. Annu. Rev. Microbiol. 72, 479–499 29932347 10.1146/annurev-micro-090817-062741

[25] Mayerle M., Raghavan M., Ledoux S., Price A., Stepankiw N., Hadjivassiliou H., Moehle E. A., Mendoza S. D., Pleiss J. A., Guthrie C., and Abelson J. (2017) Structural toggle in the RNaseH domain of Prp8 helps balance splicing fidelity and catalytic efficiency. Proc. Natl. Acad. Sci. U.S.A. 114, 4739–4744 28416677 10.1073/pnas.1701462114 PMC5422793

[26] Sibley L. D. (2004) Intracellular parasite invasion strategies. Science 304, 248–253 15073368 10.1126/science.1094717

[27] Alexander D. L., Mital J., Ward G. E., Bradley P., and Boothroyd J. C. (2005) Identification of the moving junction complex of *Toxoplasma gondii*: a collaboration between distinct secretory organelles. PLoS Pathog. 1, e17 16244709 10.1371/journal.ppat.0010017 PMC1262624

[28] David C. N., Frias E. S., Szu J. I., Vieira P. A., Hubbard J. A., Lovelace J., Michael M., Worth D., McGovern K. E., Ethell I. M., Stanley B. G., Korzus E., Fiacco T. A., Binder D. K., and Wilson E. H. (2016) GLT-1-Dependent Disruption of CNS Glutamate Homeostasis and Neuronal Function by the Protozoan Parasite *Toxoplasma gondii*. PLoS Pathog. 12, e1005643 27281462 10.1371/journal.ppat.1005643 PMC4900626

[29] Zhang N. Z., Xu Y., Wang M., Petersen E., Chen J., Huang S. Y., and Zhu X. Q. (2015) Protective efficacy of two novel DNA vaccines expressing *Toxoplasma gondii* rhomboid 4 and rhomboid 5 proteins against acute and chronic toxoplasmosis in mice. Expert Rev Vaccines 14, 1289–1297 26111968 10.1586/14760584.2015.1061938

[30] Foroutan M., and Ghaffarifar F. (2018) Calcium-dependent protein kinases are potential targets for *Toxoplasma gondii* vaccine. Clin. Exp. Vaccine Res 7, 24–36 29399577 10.7774/cevr.2018.7.1.24 PMC5795042

[31] Nitzsche R., Zagoriy V., Lucius R., and Gupta N. (2016) Metabolic cooperation of glucose and glutamine is essential for the lytic cycle of obligate intracellular parasite *Toxoplasma gondii*. J. Biol. Chem. 291, 126–141 26518878 10.1074/jbc.M114.624619 PMC4697150

[32] Kaasch A. J., and Joiner K. A. (2000) Targeting and subcellular localization of *Toxoplasma gondii* catalase. Identification of peroxisomes in an apicomplexan parasite. J. Biol. Chem. 275, 1112–1118 10625653 10.1074/jbc.275.2.1112

[33] Doll S., and Burlingame A. L. (2015) Mass spectrometry-based detection and assignment of protein posttranslational modifications. ACS Chem. Biol. 10, 63–71 25541750 10.1021/cb500904b PMC4301092

[34] Tan M., Luo H., Lee S., Jin F., Yang J. S., Montellier E., Buchou T., Cheng Z., Rousseaux S., Rajagopal N., Lu Z., Ye Z., Zhu Q., Wysocka J., Ye Y., Khochbin S., Ren B., and Zhao Y. (2011) Identification of 67 histone marks and histone lysine crotonylation as a new type of histone modification. Cell 146, 1016–1028 21925322 10.1016/j.cell.2011.08.008 PMC3176443

[35] Xu W., Wan J., Zhan J., Li X., He H., Shi Z., and Zhang H. (2017) Global profiling of crotonylation on non-histone proteins. Cell Res 27, 946–949 28429772 10.1038/cr.2017.60 PMC5518986

[36] Wei W., Mao A., Tang B., Zeng Q., Gao S., Liu X., Lu L., Li W., Du J. X., Li J., Wong J., and Liao L. (2017) Large-scale identification of protein crotonylation reveals its role in multiple cellular functions. J Proteome Res. 16, 1743–1752 28234478 10.1021/acs.jproteome.7b00012

[37] Sun H., Liu X., Li F., Li W., Zhang J., Xiao Z., Shen L., Li Y., Wang F., and Yang J. (2017) First comprehensive proteome analysis of lysine crotonylation in seedling leaves of Nicotiana tabacum. Sci. Reports 7, 3013 10.1038/s41598-017-03369-6 PMC5462846 28592803

[38] Dai L., Peng C., Montellier E., Lu Z., Chen Y., Ishii H., Debernardi A., Buchou T., Rousseaux S., Jin F., Sabari B. R., Deng Z., Allis C. D., Ren B., Khochbin S., and Zhao Y. (2014) Lysine 2-hydroxyisobutyrylation is a widely distributed active histone mark. Nat. Chem. Biol. 10, 365–370 24681537 10.1038/nchembio.1497

[39] Meng X., Xing S., Perez L. M., Peng X., Zhao Q., Redona E. D., Wang C., and Peng Z. (2017) Proteome-wide analysis of lysine 2-hydroxyisobutyrylation in developing rice (Oryza sativa) seeds. Sci. Reports 7, 17486 10.1038/s41598-017-17756-6 PMC5727541 29235492

[40] Huang H., Sabari B. R., Garcia B. A., Allis C. D., and Zhao Y. (2014) SnapShot: histone modifications. Cell 159, 458–458 e451 25303536 10.1016/j.cell.2014.09.037 PMC4324475

[41] Dong H., Guo Z., Feng W., Zhang T., Zhai G., Palusiak A., Rozalski A., Tian S., Bai X., Shen L., Chen P., Wang Q., Fan E., Cheng Z., and Zhang K. (2018) Systematic identification of lysine 2-hydroxyisobutyrylated proteins in Proteus mirabilis. Mol. Cell. Proteomics 17, 482–494, 29298837 10.1074/mcp.RA117.000430 PMC5836373

[42] Liu S., Xue C., Fang Y., Chen G., Peng X., Zhou Y., Chen C., Liu G., Gu M., Wang K., Zhang W., Wu Y., and Gong Z. (2018) Global involvement of lysine crotonylation in protein modification and transcription regulation in rice. Mol. Cell. Proteomics 17, 1922–1936 30021883 10.1074/mcp.RA118.000640 PMC6166680

[43] Yu Z., Ni J., Sheng W., Wang Z., and Wu Y. (2017) Proteome-wide identification of lysine 2-hydroxyisobutyrylation reveals conserved and novel histone modifications in Physcomitrella patens. Sci. Reports 7, 15553 10.1038/s41598-017-15854-z PMC5686104 29138512

[44] Yang Q., Li Y., Apaliya M. T., Zheng X., Serwah B. N. A., Zhang X., and Zhang H. (2018) The response of Rhodotorula mucilaginosa to patulin based on lysine crotonylation. Front. Microbiol. 9, 2025 30233516 10.3389/fmicb.2018.02025 PMC6129574

[45] Kwon O. K., Kim S. J., and Lee S. (2018) First profiling of lysine crotonylation of myofilament proteins and ribosomal proteins in zebrafish embryos. Sci. Reports 8, 3652 10.1038/s41598-018-22069-3 PMC5827021 29483630

[46] Sun J., Qiu C., Qian W., Wang Y., Sun L., Li Y., and Ding Z. (2019) Ammonium triggered the response mechanism of lysine crotonylome in tea plants. BMC Genomics 20, 340 31060518 10.1186/s12864-019-5716-z PMC6501322

[47] Liu K., Yuan C., Li H., Chen K., Lu L., Shen C., and Zheng X. (2018) A qualitative proteome-wide lysine crotonylation profiling of papaya (*Carica papaya L.*). Sci. Reports 8, 8230 10.1038/s41598-018-26676-y PMC5974297 29844531

[48] Saeij J. P. J., Boyle J. P., and Boothroyd J. C. (2005) Differences among the three major strains of *Toxoplasma gondii* and their specific interactions with the infected host. Trends Parasitol. 21, 476–481 16098810 10.1016/j.pt.2005.08.001

[49] Jeffers V., and Sullivan W. J. (2012) Lysine acetylation is widespread on proteins of diverse function and localization in the protozoan parasite *Toxoplasma gondii*. Eukaryotic Cell 11, 735–742 22544907 10.1128/EC.00088-12 PMC3370464

[50] Xue B., Jeffers V., Sullivan W. J., and Uversky V. N. (2013) Protein intrinsic disorder in the acetylome of intracellular and extracellular *Toxoplasma gondii*. Mol. Biosyst 9, 645–657 23403842 10.1039/c3mb25517d PMC3594623

[51] Caballero M. C., Alonso A. M., Deng B., Attias M., de Souza W., and Corvi M. M. (2016) Identification of new palmitoylated proteins in *Toxoplasma gondii*. Biochim. Biophys. Acta 1864, 400–408 26825284 10.1016/j.bbapap.2016.01.010 PMC4857766

[52] Foe I. T., Child M. A., Majmudar J. D., Krishnamurthy S., van der Linden W. A., Ward G. E., Martin B. R., and Bogyo M. (2015) Global analysis of palmitoylated proteins in *Toxoplasma gondii*. Cell Host Microbe 18, 501–511 26468752 10.1016/j.chom.2015.09.006 PMC4694575

[53] He C., Chen A. Y., Wei H. X., Feng X. S., and Peng H. J. (2017) Phosphoproteome of *Toxoplasma gondii* infected host cells reveals specific cellular processes predominating in different phases of infection. Am. J. Trop. Med. Hyg. 97, 236–244 28719319 10.4269/ajtmh.16-0901 PMC5508905

[54] Li X., Hu X., Wan Y., Xie G., Li X., Chen D., Cheng Z., Yi X., Liang S., and Tan F. (2014) Systematic identification of the lysine succinylation in the protozoan parasite *Toxoplasma gondii*. J. Proteome Res. 13, 6087–6095 25377623 10.1021/pr500992r

[55] Treeck M., Sanders J. L., Elias J. E., and Boothroyd J. C. (2011) The phosphoproteomes of Plasmodium falciparum and *Toxoplasma gondii* reveal unusual adaptations within and beyond the parasites' boundaries. Cell Host Microbe 10, 410–419 22018241 10.1016/j.chom.2011.09.004 PMC3254672

[56] Frenal K., Kemp L. E., and Soldati-Favre D. (2014) Emerging roles for protein S-palmitoylation in *Toxoplasma* biology. Int. J. Parasitol. 44, 121–131 24184909 10.1016/j.ijpara.2013.09.004

[57] Yakubu R. R., Silmon de Monerri N. C., Nieves E., Kim K., and Weiss L. M. (2017) Comparative monomethylarginine proteomics suggests that protein arginine methyltransferase 1 (PRMT1) is a significant contributor to arginine monomethylation in *Toxoplasma gondii*. Mol. Cell. Proteomics 16, 567–580 28143887 10.1074/mcp.M117.066951 PMC5383779

[58] Wei W., Liu X., Chen J., Gao S., Lu L., Zhang H., Ding G., Wang Z., Chen Z., Shi T., Li J., Yu J., and Wong J. (2017) Class I histone deacetylases are major histone decrotonylases: evidence for critical and broad function of histone crotonylation in transcription. Cell Res. 27, 898–915 28497810 10.1038/cr.2017.68 PMC5518989

[59] Li Y., Sabari B. R., Panchenko T., Wen H., Zhao D., Guan H., Wan L., Huang H., Tang Z., Zhao Y., Roeder R. G., Shi X., Allis C. D., and Li H. (2016) Molecular coupling of histone crotonylation and active transcription by AF9 YEATS domain. Mol. Cell 62, 181–193 27105114 10.1016/j.molcel.2016.03.028 PMC4841940

[60] Hurtley S. M. (2018) Mitochondria fight *Toxoplasma* for fat. Science 360, 504–505

[61] Lamarque M. H., Roques M., Kong-Hap M., Tonkin M. L., Rugarabamu G., Marq J. B., Penarete-Vargas D. M., Boulanger M. J., Soldati-Favre D., and Lebrun M. (2014) Plasticity and redundancy among AMA-RON pairs ensure host cell entry of *Toxoplasma* parasites. Nat. Commun. 5, 4098 24934579 10.1038/ncomms5098

[62] Frenal K., Dubremetz J. F., Lebrun M., and Soldati-Favre D. (2017) Gliding motility powers invasion and egress in Apicomplexa. Nat. Rev. Microbiol. 15, 645–660 28867819 10.1038/nrmicro.2017.86

[63] Shen B, B. J. S., Lee T D, et al (2014) Functional analysis of rhomboid proteases during *Toxoplasma* invasion. MBio 5, e01795–14. doi: 10.1128/mBio.01795-14 25336455 PMC4212836

[64] Dowse T. J., Pascall J. C., Brown K. D., and Soldati D. (2005) Apicomplexan rhomboids have a potential role in microneme protein cleavage during host cell invasion. Int. J. Parasitol. 35, 747–756 15913633 10.1016/j.ijpara.2005.04.001

[65] Zhou X. W., Blackman M. J., Howell S. A., and Carruthers V. B. (2004) Proteomic analysis of cleavage events reveals a dynamic two-step mechanism for proteolysis of a key parasite adhesive complex. Mol. Cell. Proteomics 3, 565–576 14982962 10.1074/mcp.M300123-MCP200

[66] Howell S. A., Hackett F., Jongco A. M., Withers-Martinez C., Kim K., Carruthers V. B., and Blackman M. J. (2005) Distinct mechanisms govern proteolytic shedding of a key invasion protein in apicomplexan pathogens. Mol. Microbiol. 57, 1342–1356 16102004 10.1111/j.1365-2958.2005.04772.x

[67] Etheridge R. D., Alaganan A., Tang K., Lou H. J., Turk B. E., and Sibley L. D. (2014) The *Toxoplasma* pseudokinase ROP5 forms complexes with ROP18 and ROP17 kinases that synergize to control acute virulence in mice. Cell Host Microbe 15, 537–550 24832449 10.1016/j.chom.2014.04.002 PMC4086214

[68] Hakimi M-A, O. P., Sibley LD. (2017) *Toxoplasma* effectors targeting host signaling and transcription. Clin Microbiol Rev 30, 615–645 28404792 10.1128/CMR.00005-17 PMC5475222

[69] Coppens I., Dunn J. D., Romano J. D., Pypaert M., Zhang H., Boothroyd J. C., and Joiner K. A. (2006) Toxoplasma gondii sequesters lysosomes from mammalian hosts in the vacuolar space. Cell 125, 261–274 16630815 10.1016/j.cell.2006.01.056

[70] El Bissati K., Suvorova E. S., Xiao H., Lucas O., Upadhya R., Ma Y., Angeletti R. H., White M. W., Weiss L. M., and Kim K. (2016) Toxoplasma gondii arginine methyltransferase 1 (PRMT1) is necessary for centrosome dynamics during tachyzoite cell division. MBio 7, e02094–15 26838719 10.1128/mBio.02094-15 PMC4742710

[71] Vonlaufen N., Naguleswaran A., Coppens I., and Sullivan W. J. Jr. (2010) MYST family lysine acetyltransferase facilitates ataxia telangiectasia mutated (ATM) kinase-mediated DNA damage response in *Toxoplasma gondii*. J. Biol. Chem. 285, 11154–11161 20159970 10.1074/jbc.M109.066134 PMC2856992

[72] Strobl J. S., Cassell M., Mitchell S. M., Reilly C. M., and Lindsay D. S. (2007) Scriptaid and suberoylanilide hydroxamic acid are histone deacetylase inhibitors with potent anti-*Toxoplasma gondii* activity in vitro. J. Parasitol. 93, 694–700 17626366 10.1645/GE-1043R.1

