# Supplementary material for: Global Lysine Crotonylation and 2-Hydroxyisobutyrylation in Phenotypically Different Toxoplasma gondii Parasites
Source: Mol Cell Proteomics. 2019 Sep 5;18(11):2207–24. doi: 10.1074/mcp.RA119.001611 (PMC6823851; doi:10.1074/mcp.RA119.001611)
Supplement: supplemental Data S1, S2 [file RA119.001611_index.html]

Supplement to Global lysine crotonylation and 2-hydroxyisobutyrylation in phenotypically different Toxoplasma gondii parasites | Molecular & Cellular Proteomics

## Supplemental Data

- Supplementary figures - Supplementary figures and legends.
- Supplementary dataset 1 - Supplementary dataset 1
- Supplementary dataset 2 - Supplementary dataset 2
- Supplementary dataset 3 - Supplementary dataset 3
- Supplementary dataset 4 - Supplementary dataset 4
- Supplementary dataset 5 - Supplementary dataset 5
- Supplementary dataset 6 - Supplementary dataset 6
- Supplementary dataset 7 - Supplementary dataset 7
- Supplementary dataset 8 - Supplementary dataset 8
- Supplementary dataset 9 - Supplementary dataset 9
- Supplementary dataset 10 - Supplementary dataset 10
- Supplementary dataset 11 - Supplementary dataset 11
- Supplementary dataset 12 - Supplementary dataset 12
- Supplementary dataset 13 - Supplementary dataset 13
- Supplementary dataset 14 - Supplementary dataset 14
- Supplementary dataset 15 - Supplementary dataset 15
- Supplementary dataset 16 - Supplementary dataset 16
- Supplementary dataset 17 - Supplementary dataset 17
- Supplementary dataset 18 - Supplementary dataset 18
- Supplementary dataset 19 - Supplementary dataset 19
- Supplementary dataset 20 - Supplementary dataset 20
- Supplementary dataset 21 - Supplementary dataset 21
- Supplementary dataset 22 - Supplementary dataset 22
- Supplementary dataset 23 - Supplementary dataset 23
