# Supplementary material for: Global Lysine Crotonylation and 2-Hydroxyisobutyrylation in Phenotypically Different Toxoplasma gondii Parasites
Source: Mol Cell Proteomics. 2019 Sep 5;18(11):2207–24. doi: 10.1074/mcp.RA119.001611 (PMC6823851; doi:10.1074/mcp.RA119.001611)
Supplement: Supplementary figures [file 153316_2_supp_389873_pxb4pw.pdf]

Supplementary information

Global lysine crotonylation and 2-hydroxyisobutyrylation in the phenotypically different *Toxoplasma gondii* parasites

Deqi Yin[1], Yue Zhang[1], Dawei Wang[1], Xiaoyu Sang[1], Ying Feng[1], Rang Chen[1], Xinyi Wang[2], Na Yang[1], Ning Jiang[1], Qijun Chen[1#]

Table of Contents

Supplementary Figures

Supplementary Figure 1: The correlation and coefficient of variation for biological replicates.

Supplementary Figure 2: Venn diagram of lysine crotonylation and 2-hydroxyisobutyrylation sites.

Supplementary Figure 3: Gene Ontology (GO) terms are significantly enriched in crotonylated and 2-hydroxyisobutyrylated proteins (CC).

Supplementary Figure 4: Gene ontology (GO) terms are significantly enriched in crotonylated and 2-hydroxyisobutyrylated proteins (MF).

Supplementary Figure 5: Gene ontology (GO) terms are significantly enriched in crotonylated and 2-hydroxyisobutyrylated proteins (BP).

Supplementary Figure 6: Characterization of crotonylated and 2-hydroxyisobutyrylated peptides.

Supplementary Figure 7: Quantitative analysis: Volcano plot of differential crotonylation sites in two *T. gondii* strains.

Supplementary Figure 8: Quantitative analysis: Volcano plot of differential 2-hydroxyisobutyrylation sites in two *T. gondii* strains.

Supplementary Figure 9: Quantitative analysis: Gene Ontology functional classification of the differentially crotonylated and 2-hydroxyisobutyrylated proteins in the category of cellular component (CC) (Fisher's exact test, $p < 0.05$).

Supplementary Figure 10: Quantitative analysis: Gene Ontology functional classification of the differentially crotonylated and 2-hydroxyisobutyrylated proteins in the category of molecular function (MF) (Fisher's exact test, $p < 0.05$).

Supplementary Figure 11: Quantitative analysis: Gene Ontology functional classification of the differentially crotonylated and 2-hydroxyisobutyrylated proteins in the category of biological process (BP) (Fisher's exact test, $p < 0.05$).

Supplementary Figure 12: Crotonylation and 2-hydroxyisobutyrylation of key enzymes involved in glycolysis/gluconeogenesis, citrate cycle (TCA cycle) and peroxisome processes.

Supplementary Figure 13: The differentially crotonylated and 2-hydroxyisobutyrylated proteins involved in aminoacyl−tRNA biosynthesis.
Supplementary Figure 14: The interaction network of differentially modified proteins.
Supplementary Figure 15: The interaction network of specifically modified proteins.

Supplementary Tables

Supplementary Table 1: The differentially modified proteins of transcriptional regulators, the spliceosomal complex and chromatin remodelling complexes.
Supplementary Table 2: The differentially modified enzymes involved in glycolysis/gluconeogenesis.
Supplementary Table 3: The differentially modified enzymes in the citrate cycle (TCA cycle) and peroxisome processes.
Supplementary Table 4: The differentially crotonylated and 2-hydroxyisobutyrylated proteins related to aminoacyl-tRNA biosynthesis.
Supplementary Table 5: The differentially crotonylated and 2-hydroxyisobutyrylated proteins involved in protein biosynthesis, folding, and ubiquitin-dependent degradation.
Supplementary Table 6: Proteomic studies of PTMs in *T. gondii.*
Supplementary Table 7: Detected modification sites on *T. gondii* histones.

Supplementary Data

Supplementary Data 1: Qualitative analysis of *T. gondii* (RH strain) proteome.
Supplementary Data 2: Qualitative analysis of *T. gondii* (ME49 strain) proteome.
Supplementary Data 3: Quantitative analysis of *T. gondii* (RH and ME49 strains) proteome.
Supplementary Data 4: Subcellular localization and GO analysis of differential proteins.
Supplementary Data 5: Qualitative analysis of Crotonylation in the phenotypically different *T. gondii* parasites.

Supplementary Data 6: Qualitative analysis of 2-hydroxyisobutyrylation in the phenotypically different *T. gondii* parasites.

Supplementary Data 7: GO enrichment analysis of crotonylated and 2-hydroxyisobutyrylated proteins.

Supplementary Data 8: KEGG enrichment analysis of crotonylated and 2-hydroxyisobutyrylated proteins.

Supplementary Data 9: Quantitative analysis of Crotonylation in *T. gondii* (RH and ME49 strains).

Supplementary Data 10: Quantitative analysis of 2-hydroxyisobutyrylation in *T. gondii* (RH and ME49 strains).

Supplementary Data 11: The differential crotonylation sites in *T. gondii.*

Supplementary Data 12: The differential 2-hydroxyisobutyrylation sites in *T. gondii.*

Supplementary Data 13: The differentially crotonylated and 2-hydroxyisobutyrylated proteins were classified based on subcellular location in *T. gondii.*

Supplementary Data 14: KOG (Eukaryotic Orthologous Groups) analysis of differentially modified proteins.

Supplementary Data 15: Gene Ontology functional classification of the differentially crotonylated and 2-hydroxyisobutyrylated proteins.

Supplementary Data 16: KEGG enrichment analysis of the differentially crotonylated proteins.

Supplementary Data 17: KEGG enrichment analysis of the differentially 2-hydroxyisobutyrylated proteins.

Supplementary Data 18: The differentially modified proteins of transcriptional regulators, the spliceosomal complex and chromatin remodelling complexes.

Supplementary Data 19: Lysine crotonylation and 2-hydroxyisobutyrylation of invasion-related proteins.

Supplementary Data 20: Crotonylation and 2-hydroxyisobutyrylation of key enzymes involved in glycolysis/gluconeogenesis, citrate cycle (TCA cycle) and peroxisome processes.

Supplementary Data 21: The differentially crotonylated and 2-hydroxyisobutyrylated proteins related to aminoacyl-tRNA biosynthesis, protein biosynthesis, folding, and ubiquitin-dependent degradation.

Supplementary Figure 22: The interaction network of differentially modified proteins.

Supplementary Figure 23: The interaction network of specifically modified proteins.

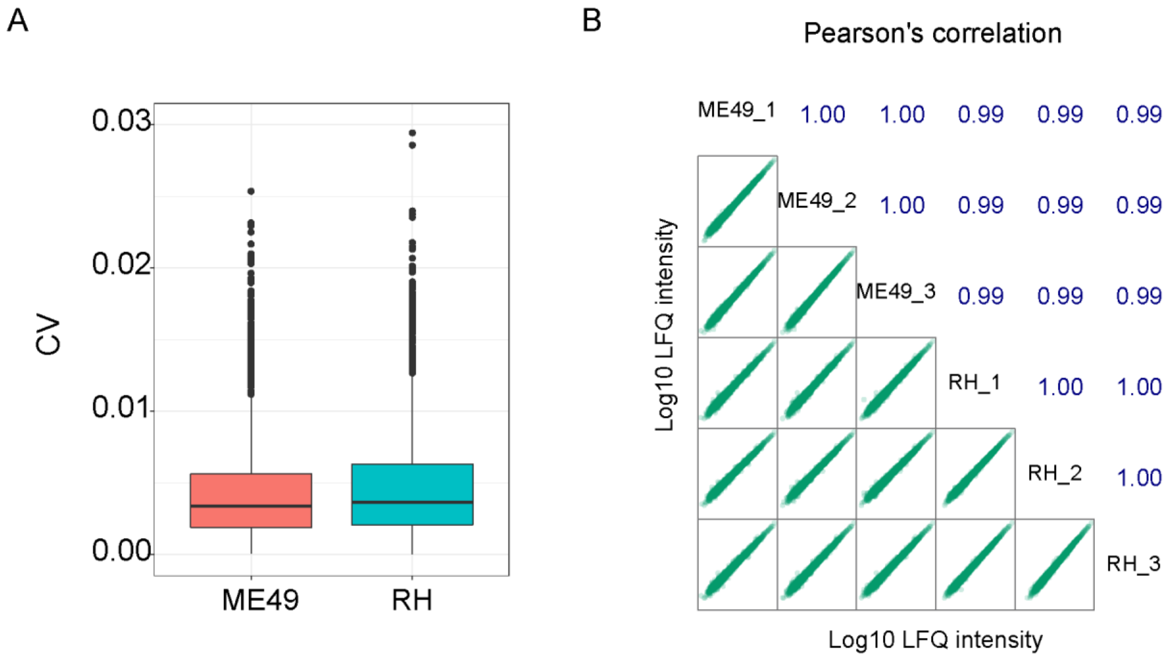

Figure S1. The correlation and coefficient of variation for biological replicates. (A) Coefficient of variation. (B) Pearson correlation coefficient of three replications.

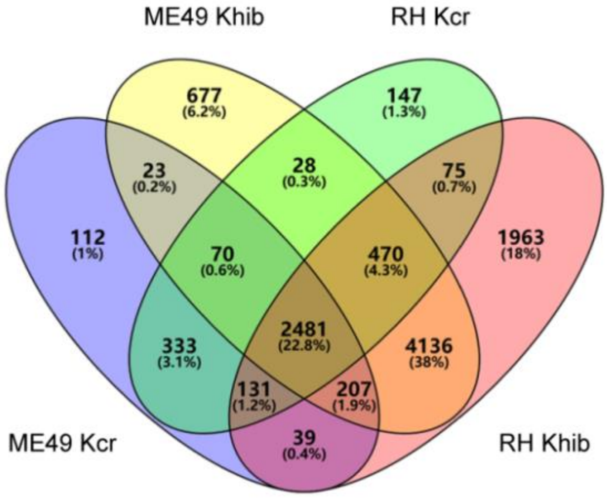

Figure S2. Venn diagram of lysine crotonylation and 2-hydroxyisobutyrylation sites. Venn diagrams representing overlap between lysine crotonylation sites and 2-hydroxyisobutyrylation sites in two *T. gondii* strains. Ellipses in green and pink represent the number of crotonylation sites and 2-hydroxyisobutyrylation sites identified in RH strain *T. gondii*, respectively. Ellipses in blue and yellow represent the number of crotonylation sites and 2-hydroxyisobutyrylation sites identified in ME49 strain *T. gondii*, respectively. Overlap regions represent the number of sites with both modifications in the two parasite strains.

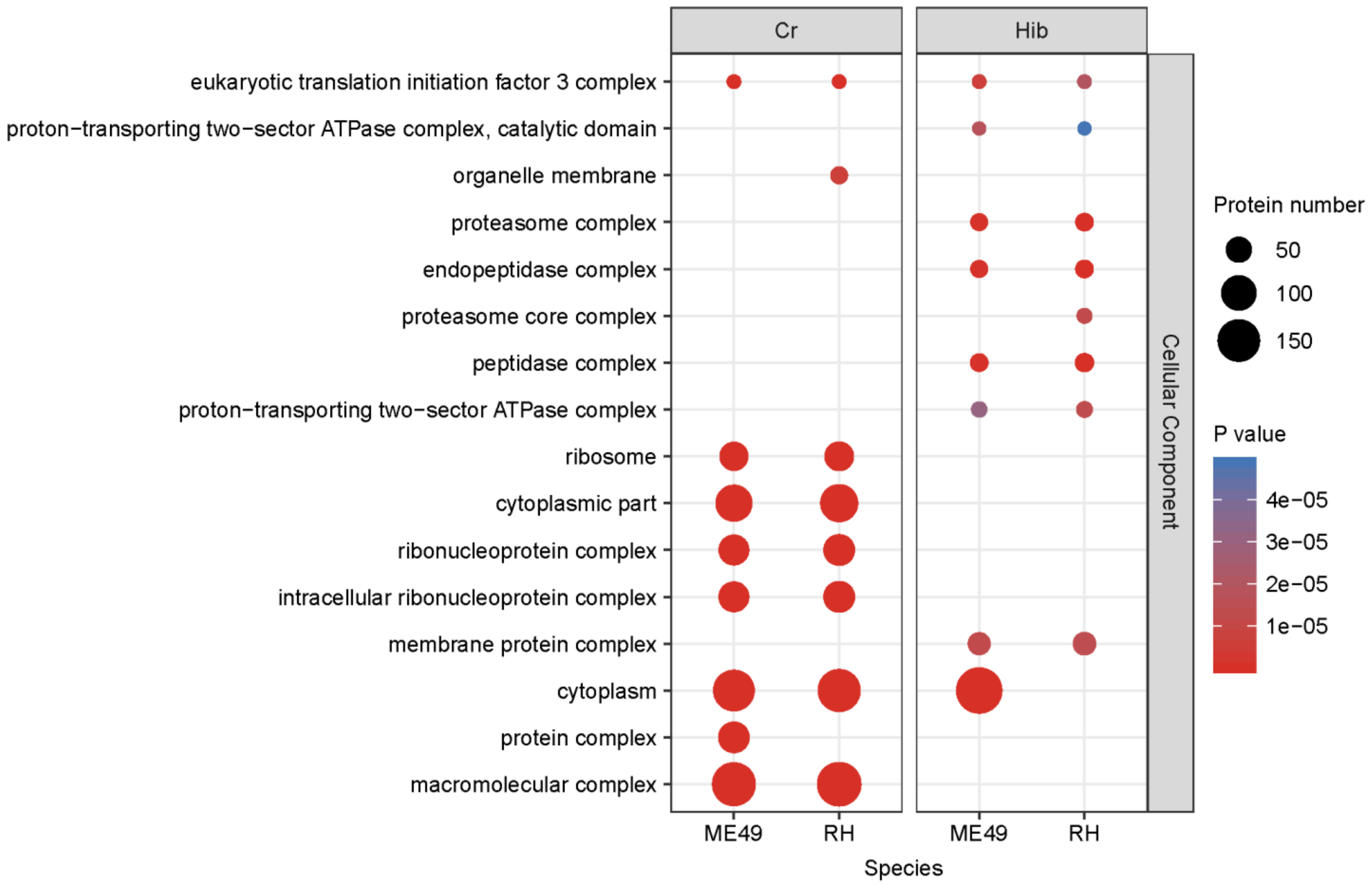

Figure S3. GO-based enrichment analysis of crotonylated and 2-hydroxyisobutyrylated proteins. Qualitative analysis: Gene Ontology functional classification of crotonylated and 2-hydroxyisobutyrylated proteins in the category of cellular component (CC) (detailed data are listed in Supplementary Data 7) (Fisher's exact test, $p < 0.05$).

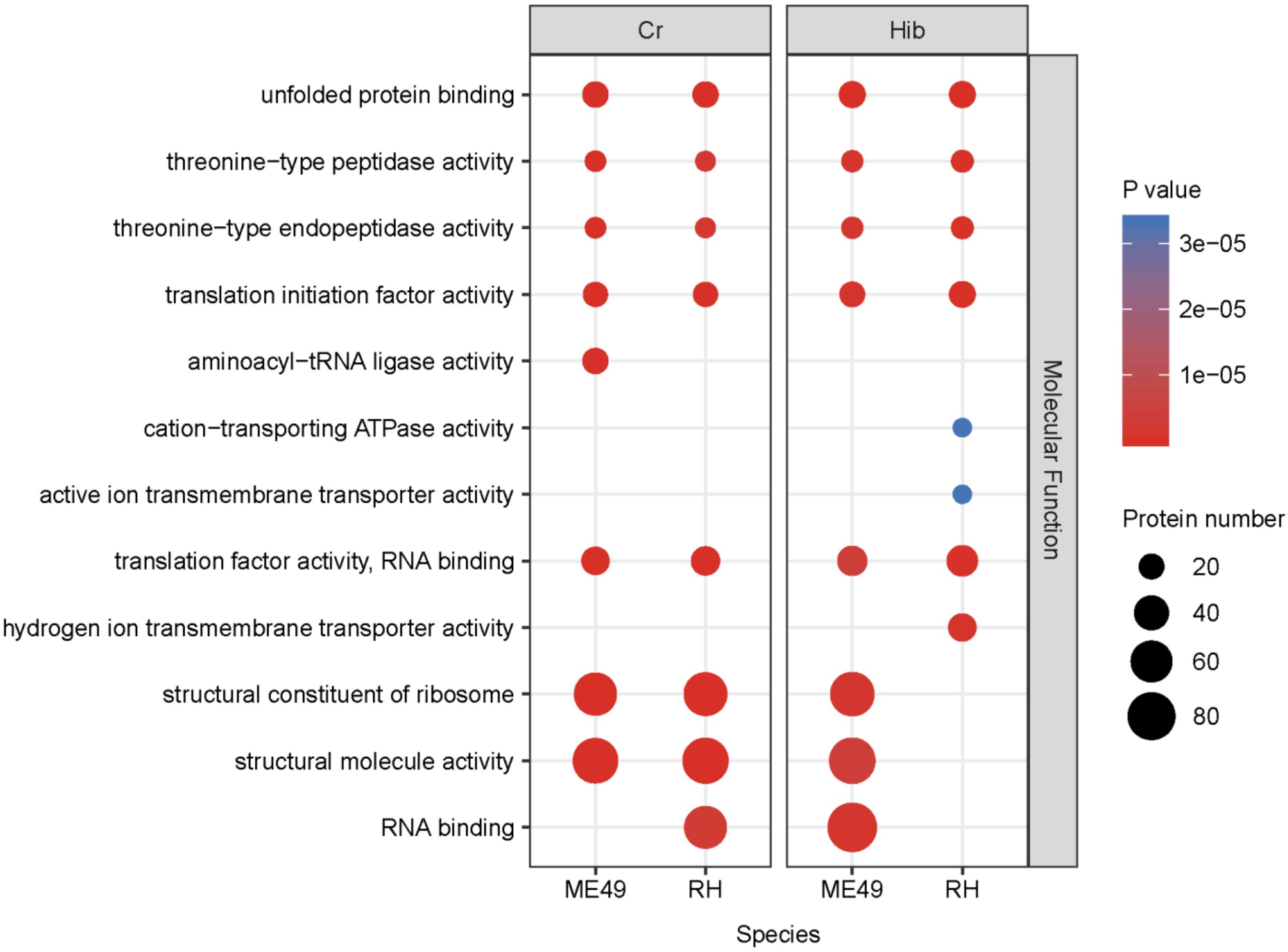

Figure S4. GO-based enrichment analysis of crotonylated and 2-hydroxyisobutyrylated proteins. Qualitative analysis: Gene Ontology functional classification of crotonylated and 2-hydroxyisobutyrylated proteins in the category of molecular function (MF) (detailed data are listed in Supplementary Data 7) (Fisher's exact test, $p < 0.05$).

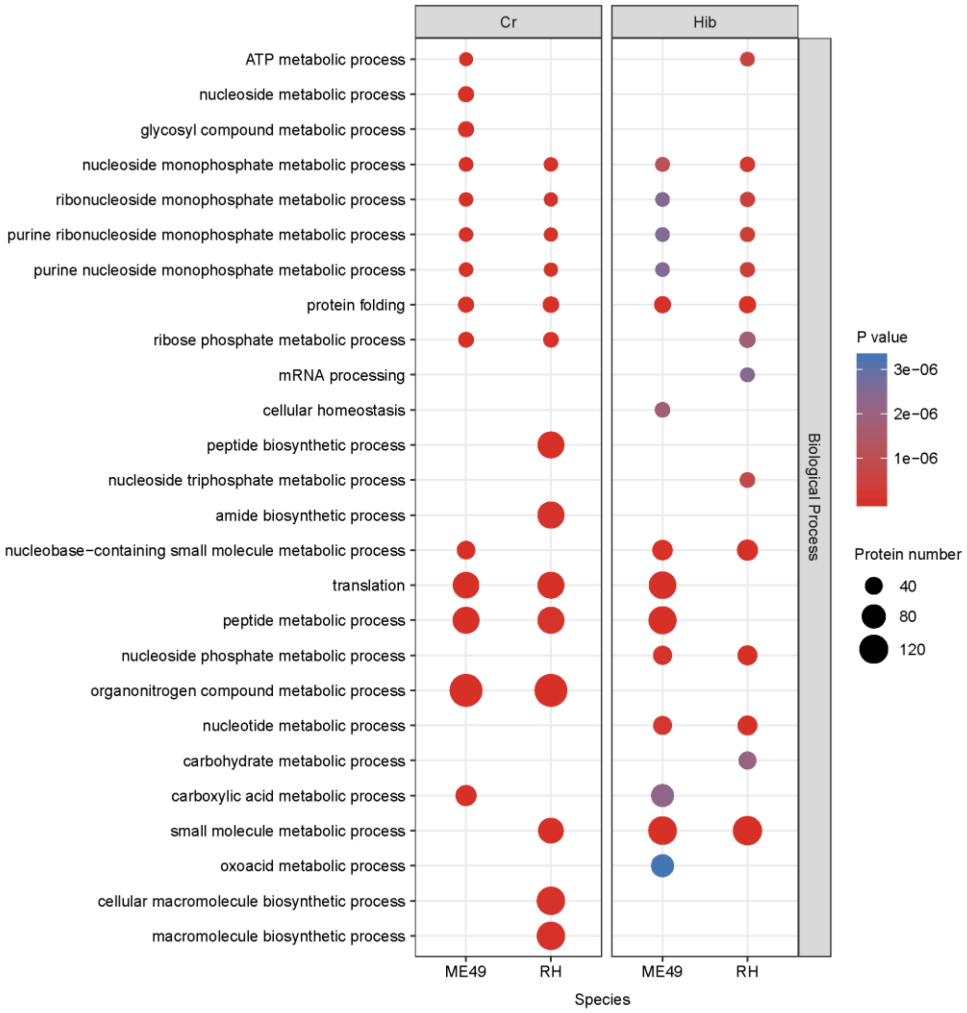

Figure S5. GO-based enrichment analysis of crotonylated and 2-hydroxyisobutyrylated proteins. Qualitative analysis: Gene Ontology functional classification of crotonylated and 2-hydroxyisobutyrylated proteins in the category of biological process (BP) (detailed data are listed in Supplementary Data 7) (Fisher's exact test, $p < 0.05$).

A

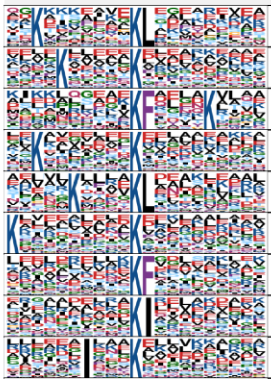

Motif Logo	Motif	Motif Score	Foreground		Background		Fold Increase
			Matches	Size	Matches	Size	
	..K.......KL.........	23.67	49	2778	513	120261	4.13
	....K.....K..........	16	292	2729	7054	119748	1.82
	..........KF....K....	23.74	23	2437	147	112694	7.24
	..K.......K..........	16	237	2414	6058	112547	1.82
	.....K....KL.........	22.55	38	2177	441	106489	4.21
	K.........K..........	16	205	2139	5422	106048	1.87
	..........KF.........	16	127	1934	2249	100626	2.94
	..........KI.........	16	125	1807	2924	98377	2.33
	......I...K..........	16	105	1682	2259	95453	2.64

B

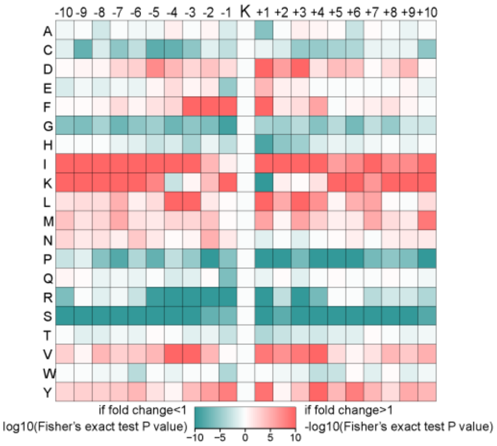

C

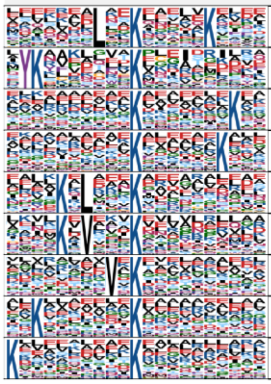

Motif Logo	Motif	Motif Score	Foreground		Background		Fold Increase
			Matches	Size	Matches	Size	
	.......L..K.....K....	23.85	98	7499	1168	257166	2.88
	.YK.......K..........	22.14	26	7401	193	255998	4.66
	..........K.......K..	16	654	7375	15345	255805	1.48
	..........K......K...	16	577	6721	13490	240460	1.53
	....K.L...K..........	23.04	78	6144	988	226970	2.92
	....K.V...K..........	22.06	54	6066	665	225982	3.03
	........V.K..........	16	502	6012	12555	225317	1.5
	..K.......K..........	16	461	5510	11822	212762	1.51
	K.........K..........	16	414	5049	10117	200940	1.63

D

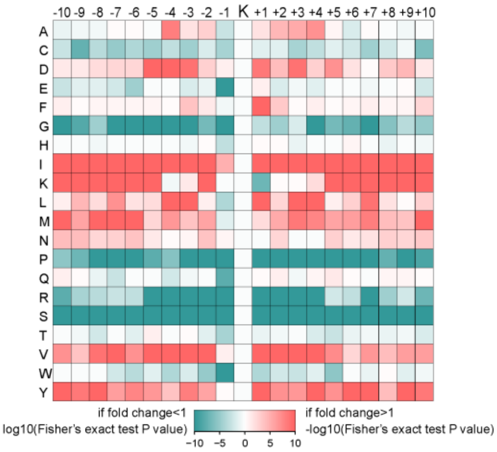

Figure S6. Characterization of crotonylated and 2-hydroxyisobutyrylated peptides. (A) Probability sequence motifs of crotonylation sites consisting of 20 residues surrounding the targeted lysine residue produced using Motif-x. Nine significantly enriched crotonylation site motifs were identified in the phenotypically different *T. gondii* parasites. (B) Heat map showing enrichment (red) or depletion (green) of amino acids in specific positions flanking the crotonylated lysine. (C) Probability sequence motifs of 2-hydroxyisobutyrylation sites consisting of 20 residues surrounding the targeted lysine residue produced using Motif-x. Nine significantly enriched 2-hydroxyisobutyrylation site motifs were identified in the phenotypically different *T. gondii* parasites. (D) Heat map showing enrichment (red) or depletion (green) of amino acids in specific positions flanking the 2-hydroxyisobutyrylated lysine.

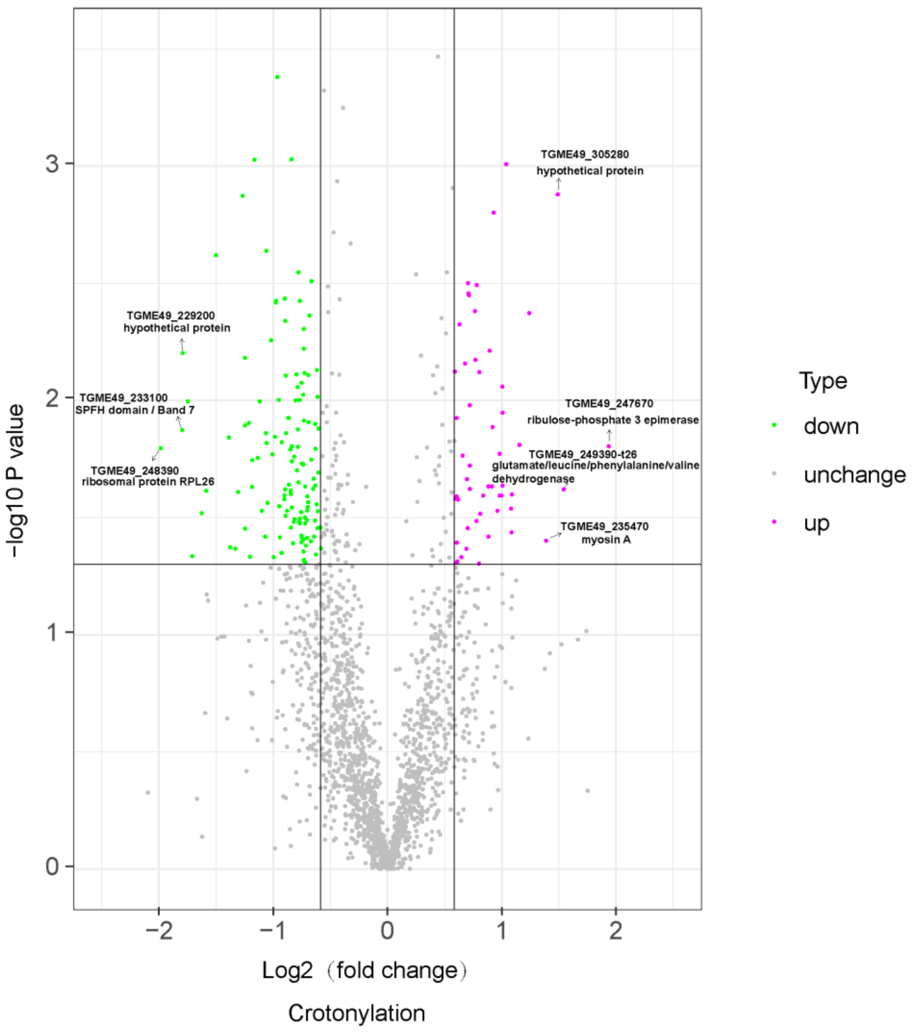

Figure S7. Quantitative analysis: Volcano plot of differential crotonylation sites in two *T. gondii* strains. Volcano plot showing differential crotonylation sites (fold change > 1.5; t test, $p < 0.05$). Magenta dots represent upregulated crotonylation sites. Green dots represent downregulated crotonylation sites (detailed data are listed in Supplementary Data 11).

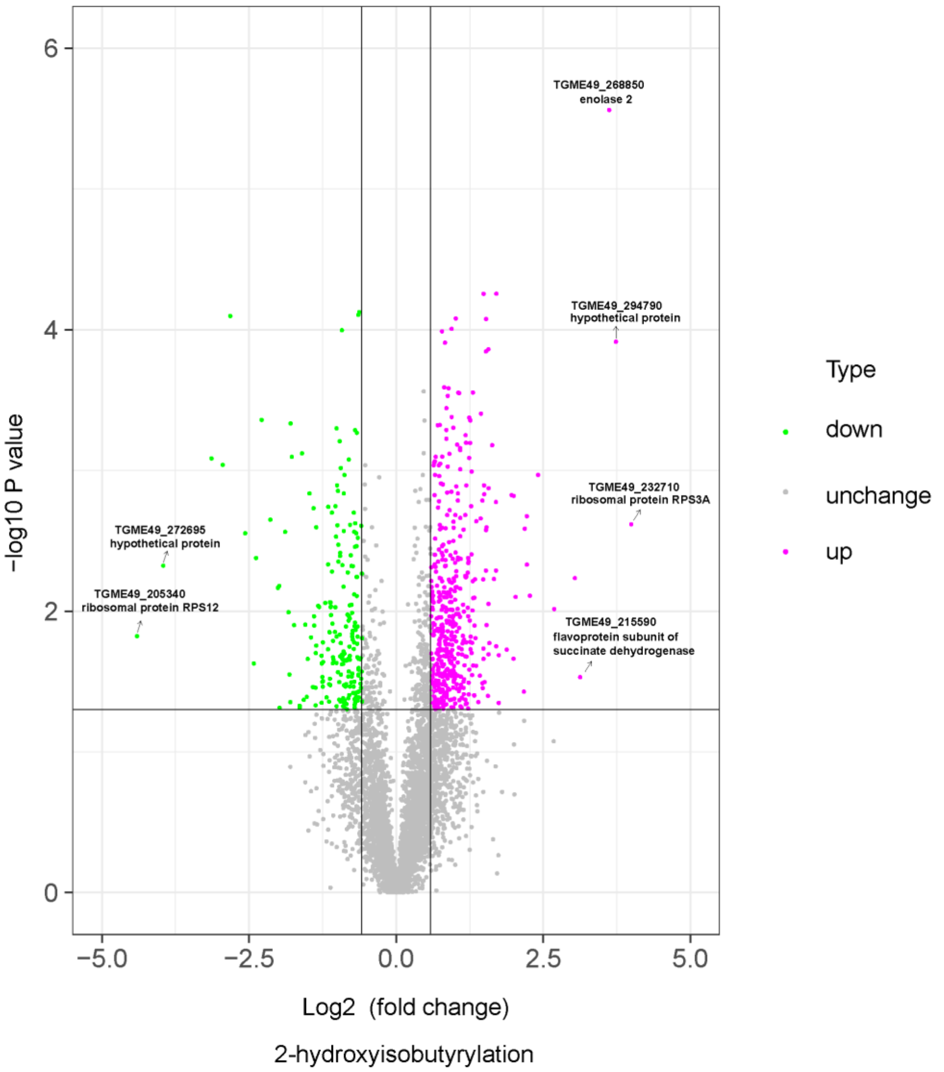

Figure S8. Quantitative analysis: Volcano plot of differential 2-hydroxyisobutyrylation sites in two *T. gondii* strains. Volcano plot showing differential 2-hydroxyisobutyrylation sites (fold change > 1.5; $p < 0.05$). Magenta dots represent upregulated 2-hydroxyisobutyrylation sites. Green dots represent downregulated 2-hydroxyisobutyrylation sites (detailed data are listed in Supplementary Data 12).

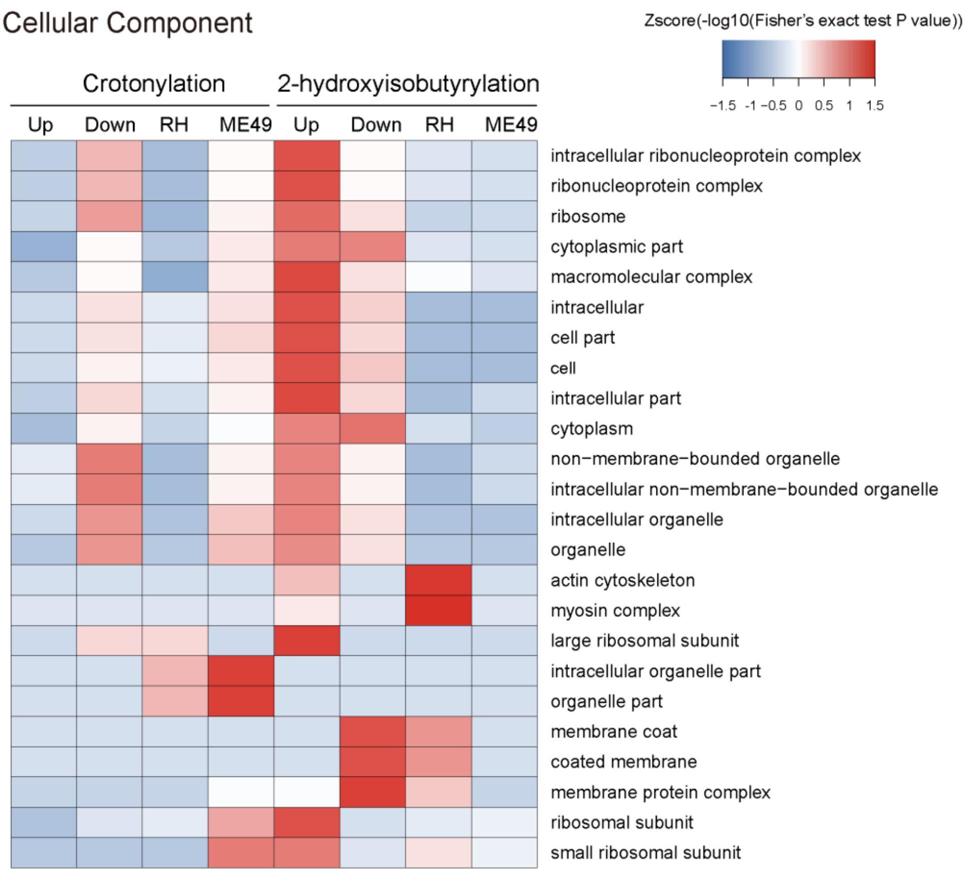

Figure S9. Quantitative analysis: Gene Ontology functional classification of differentially crotonylated and 2-hydroxyisobutyrylated proteins in the category of cellular component (CC) (Fisher's exact test, *p* < 0.05) (detailed data are listed in Supplementary Data 15).

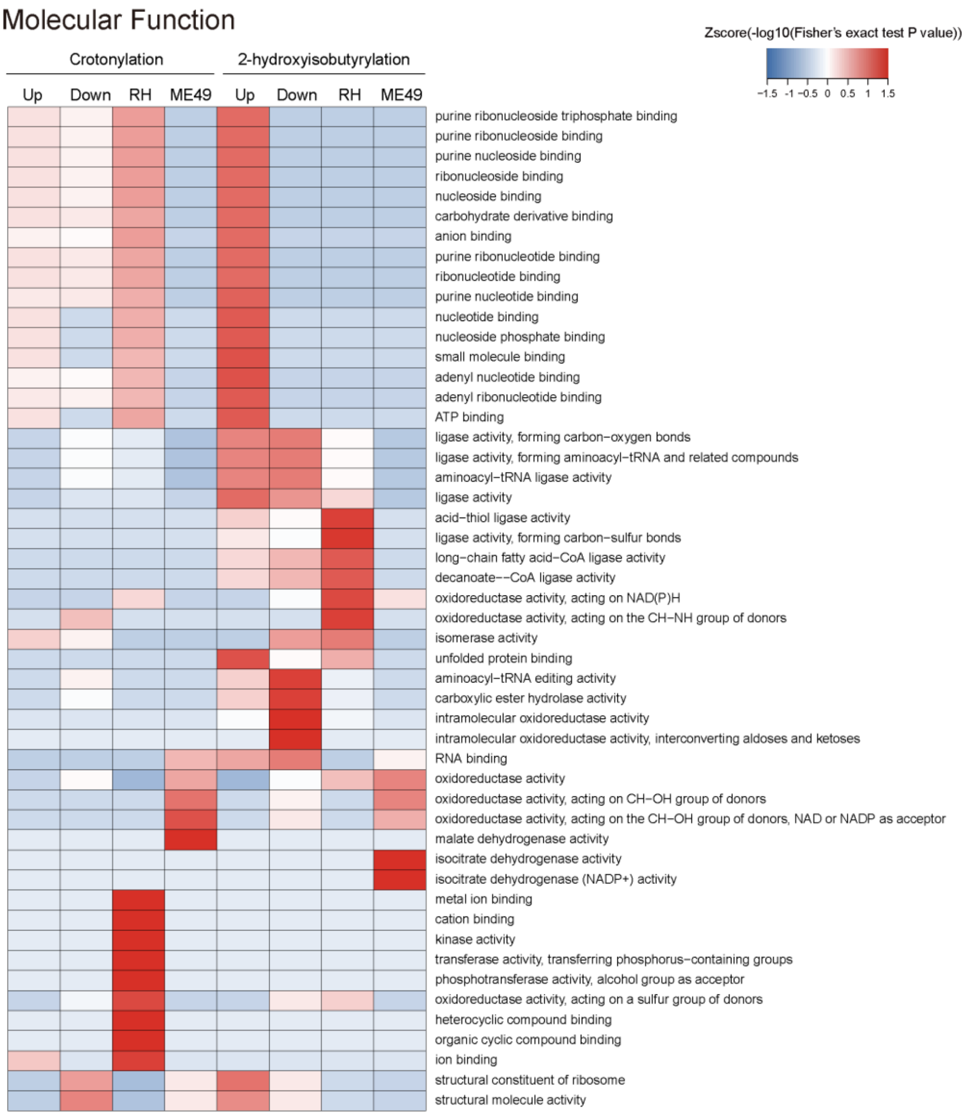

Figure S10. Quantitative analysis: Gene Ontology functional classification of differentially crotonylated and 2-hydroxyisobutyrylated proteins in the category of molecular function (MF) (Fisher's exact test, *p* < 0.05) (detailed data are listed in Supplementary Data 15).

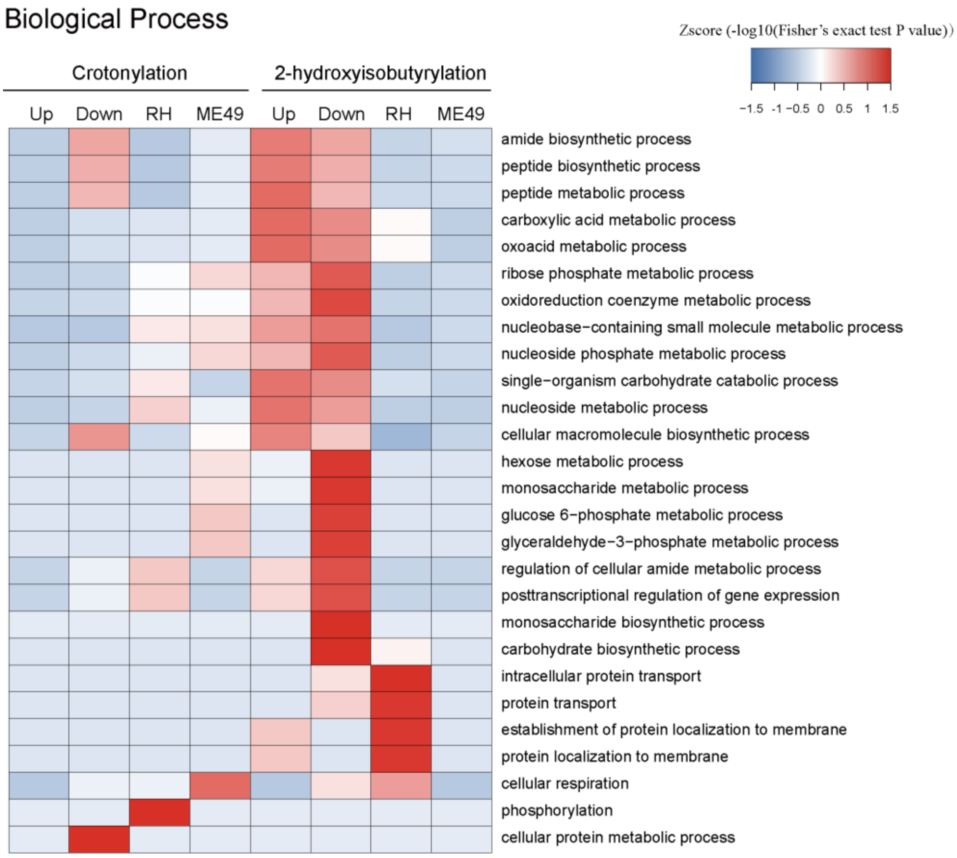

Figure S11. Quantitative analysis: Gene Ontology functional classification of differentially crotonylated and 2-hydroxyisobutyrylated proteins in the category of biological process (BP) (Fisher's exact test, $p < 0.05$) (detailed data are listed in Supplementary Data 15).

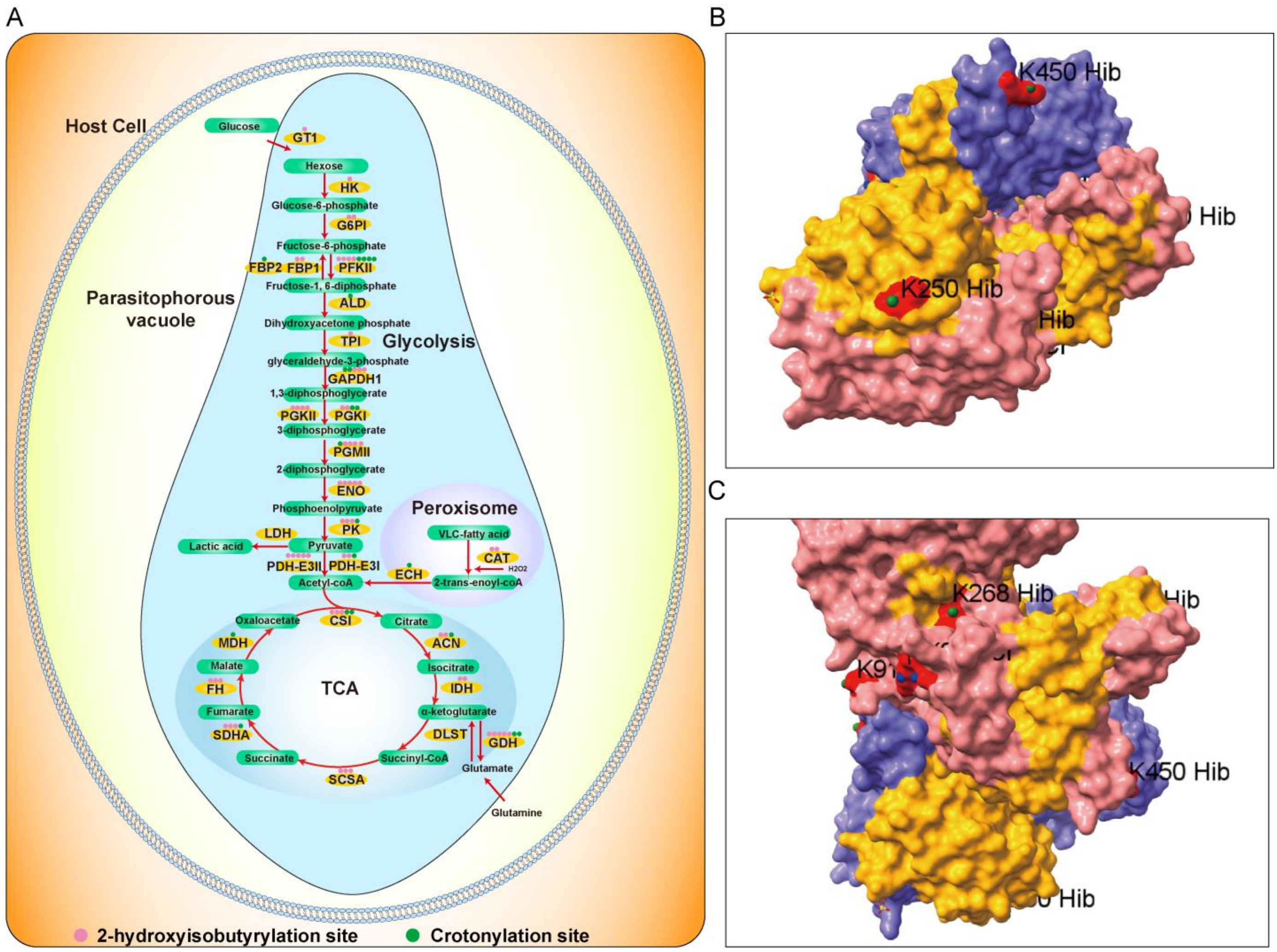

Figure S12. Crotonylation and 2-hydroxyisobutyrylation of key enzymes involved in glycolysis/gluconeogenesis, citrate cycle (TCA cycle) and peroxisome processes. (A) The differentially crotonylated and 2-hydroxyisobutyrylated proteins involved in glycolysis/gluconeogenesis, citrate cycle (TCA cycle) and peroxisome processes. (B) Lysine 2-hydroxyisobutyrylation sites in the structure of the phosphofructokinase domain (K250, 2-hydroxyisobutyrylation). A representative structure pertaining to Protein Data Bank (PDB) ID 1KZH is shown. The yellow structure is the active region of phosphofructokinase. (C) Lysine 2-hydroxyisobutyrylation sites in the structure of the phosphofructokinase domain (K268, 2-hydroxyisobutyrylation). A representative structure pertaining to Protein Data Bank (PDB) ID 1KZH is shown. The yellow structure is the active region of phosphofructokinase.

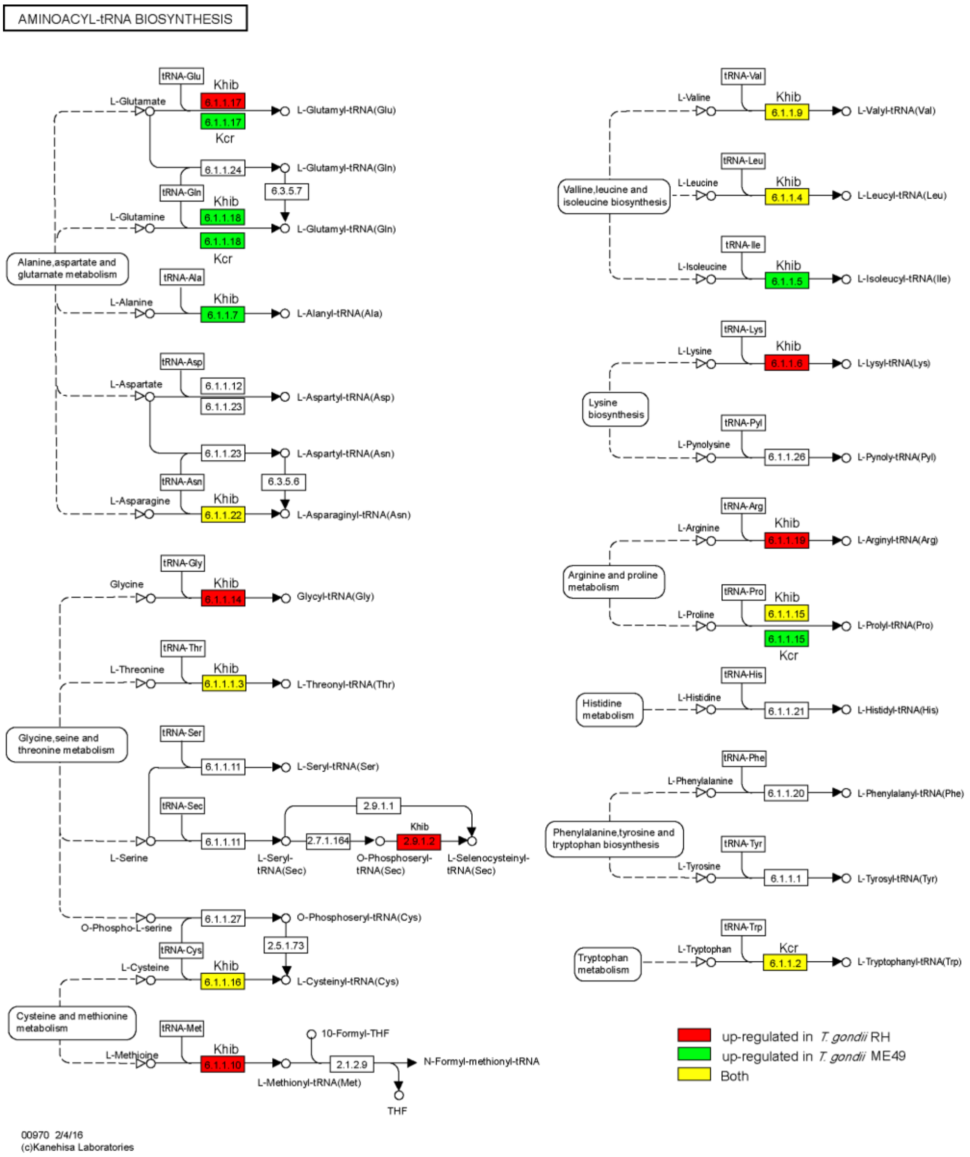

Figure S13. The differentially crotonylated and 2-hydroxyisobutyrylated proteins involved in aminoacyl−tRNA biosynthesis. Red boxes represent the proteins with upregulated modifications. Green boxes represent the proteins with downregulated modifications. Yellow boxes represent proteins with both upregulated and downregulated modifications. The number in the box represents the number of the enzyme (Enzyme Commission numbers). Detailed data are listed in Supplementary Table 4.

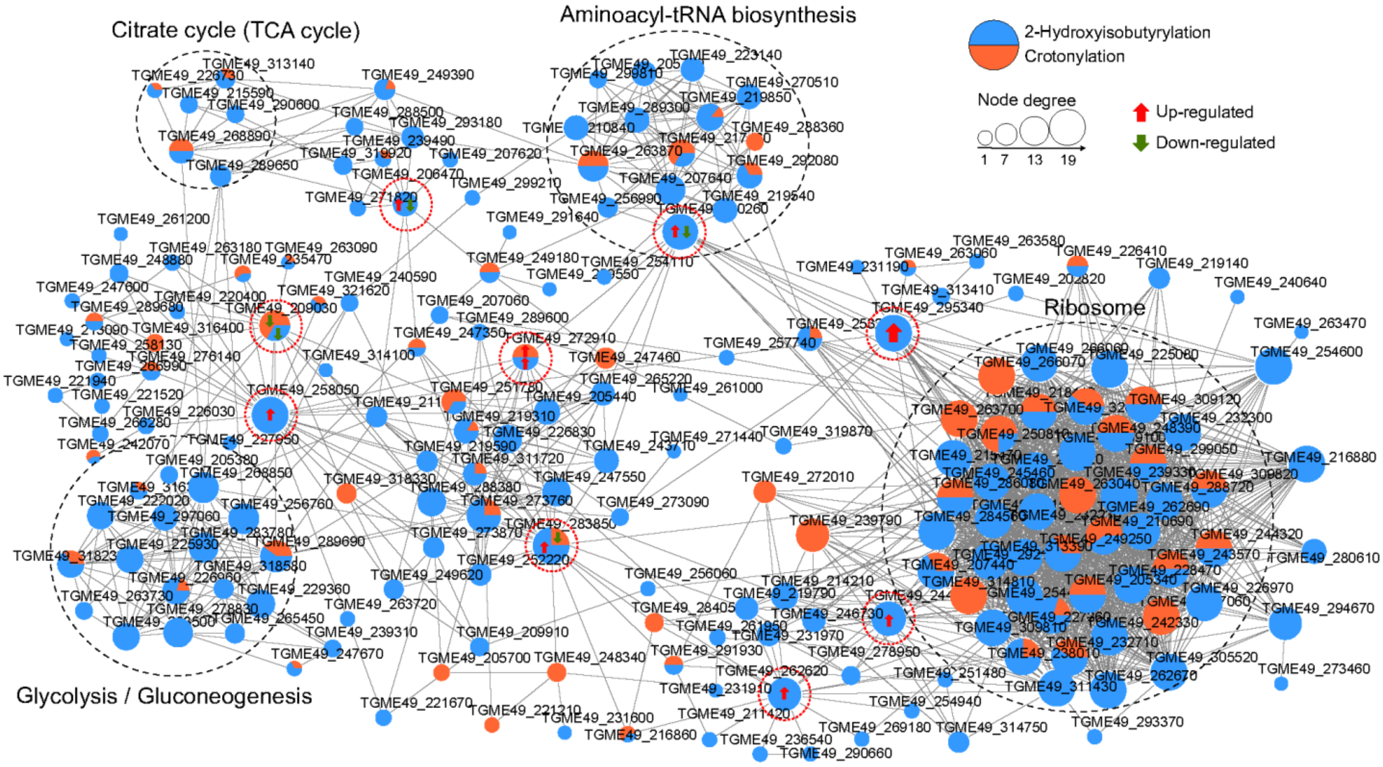

Figure S14. The interaction network of differentially modified proteins listed by gene names, based on the STRING database and visualized in Cytoscape (detailed data are listed in Supplementary Data 22).

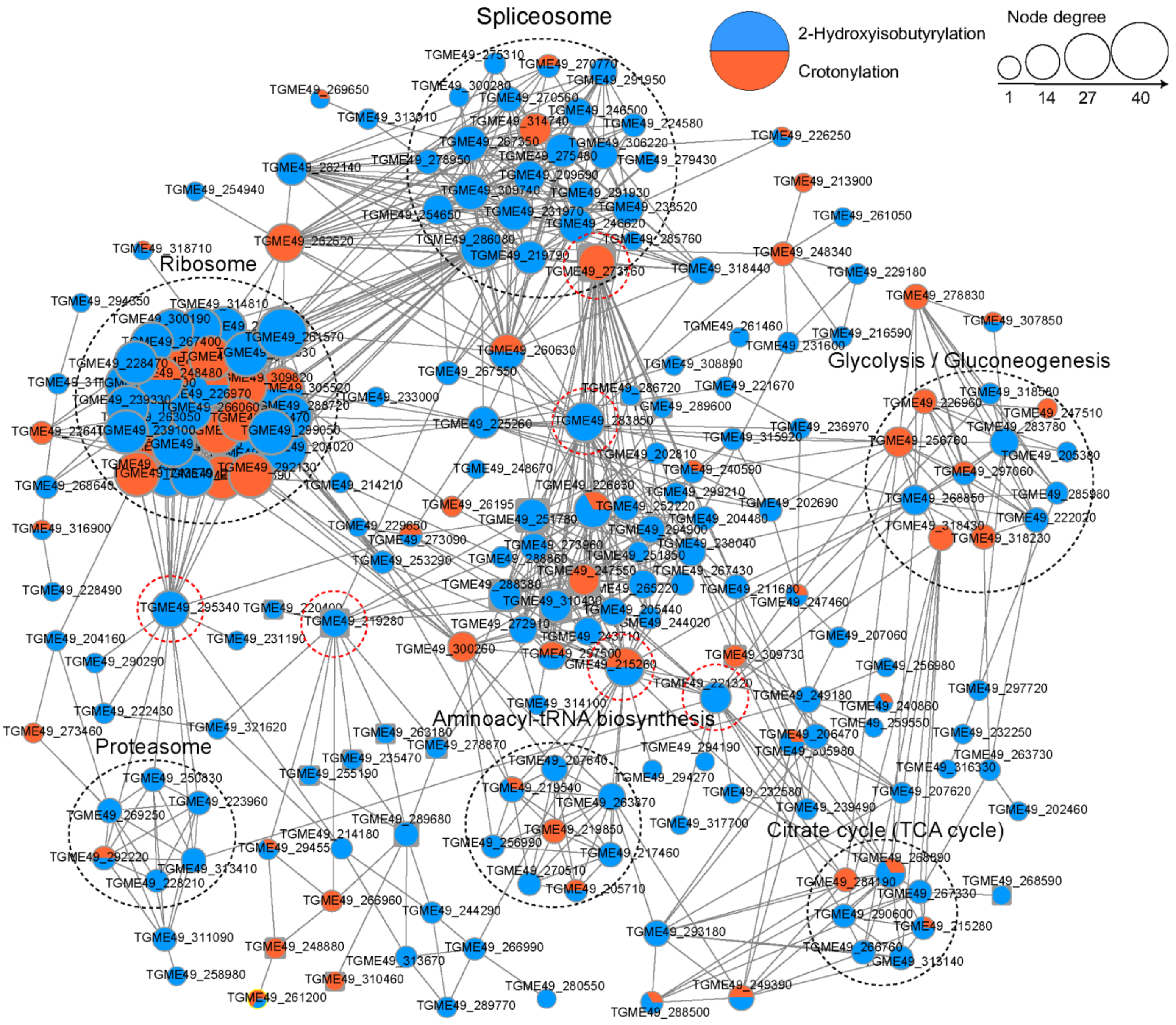

Figure S15. The interaction network of specifically modified proteins listed by gene names, based on the STRING database and visualized in Cytoscape (detailed data are listed in Supplementary Data 23).

Table S1: The differentially modified proteins of transcriptional regulators, the spliceosomal complex and chromatin remodelling complexes

	Crotonylation			2-hydroxyisobutyrylation		
**Type**	**Up-regulated**	**Down-regulated**	**Specific**	**Up-regulated**	**Down-regulated**	**Specific**
**Histone modification enzyme**						
PRMT2				K935		
HDAC2				K1,226		
PRMT4/CARM1						K1,689(RH)
MYST-A		K54				
**DNA-directed RNA polymerase**						
RPB1				K726		K279(RH)
RPB5			K726(RH)	K984		K945(RH), K958(RH)
RPB9						K179(RH)
RPB11A				K544		K576(RH), K1,979(RH)
**Elongation factors**						
transcriptional elongation factor FACT80						K104(RH)
transcriptional elongation factor FACT140					K295	K226(RH)<br>K375(RH)
elongation factor 2 family protein		K1,430		K542		K339(ME49)
transcription elongation factor SPT6						K2,360(RH)
**Pre-mRNA splicing**						
PRP8				K1,508	K757, K2,315	K526(RH), K644(RH), K1,055(RH)
cwf18						K105(RH)
splicing factor U2AF				K566		
FUSE-binding protein 2/KH-type KSRP			K761(RH)	K807	K660	
(RH) represents a specific modification site of *T. gondii* RH strain. (ME49) represents a specific modification site of *T. gondii* ME49 strain (detailed data are listed in Supplementary Data 18).						

Table S2: The differentially modified enzymes involved in glycolysis/gluconeogenesis

	Crotonylation			2-hydroxyisobutyrylation		
**Proteins**	**Up-regulated**	**Down-regulated**	**Specific**	**Up-regulated**	**Down-regulated**	**Specific**
Aldehyde dehydrogenase ALD			K401(ME49)			
Facilitative glucose transporter				K563		
Hexokinase				K304		
Fructose-bisphospatase II FBPII			K70(RH)			
Triose-phosphate isomerase				K58		
Fructose-bisphospatase I FBPI				K140		K385(RH)
Glucose-6-phosphate isomerase					K212	K154(RH)
Pyruvate dehydrogenase E1 component subunit beta PDH-E3I			K377(RH)			K447(ME49) K631(RH)
Phosphoglycerate kinase PGKI	K264		K191(RH)	K131, K149		
Phosphoglycerate kinase PGKII				K313, K404	K243	K210(ME49)
Pyruvate kinase PyK1			K1,177(RH)	K228, K310	K219	
Pyruvate dehydrogenase complex subunit PDH-E3II				K176, K276	K286	K450(RH) K505(RH)
Enolase 2				K118, K162, K449		K92 (RH) K308(RH)
Phosphoglycerate mutase PGMII			K244(RH)	K195, K337	K132	K161(RH)
Glyceraldehyde-3-phosphate dehydrogenase GAPDH1		K211, K274		K214	K253, K274	
phosphofructokinase PFKII		K928	K861(RH) K951(ME49) K1,209(RH)	K250, K268	K450, K914	

The modified enzymes included facilitative glucose transporter GT1 (GT1), hexokinase (HK), glucose-6-phosphate isomerase GPI (G6PI), fructose-bisphospatase I (FBP I), fructose-bisphospatase II (FBP II), phosphofructokinase PFKII, aldehyde dehydrogenase (ALD), triose-phosphate isomerase TPI-I, phosphoglycerate kinase PGKII, phosphoglycerate kinase PGKI, glyceraldehyde-3-phosphate dehydrogenase GAPDH1, phosphoglycerate mutase PGMII, enolase 2 (ENO), pyruvate kinase PyK1 (PK), lactate dehydrogenase (LDH), pyruvate dehydrogenase E1 component subunit beta (PDH-E3I), pyruvate dehydrogenase complex subunit (PDH-E3II). (RH) represents a specific modification site of *T. gondii* RH strain. (ME49) represents a specific modification site of *T. gondii* ME49 strain (detailed data are listed in Supplementary Data 20).

Table S3: The differentially modified enzymes in the citrate cycle (TCA cycle) and peroxisome processes

	Crotonylation			2-hydroxyisobutyrylation		
**Proteins**	**Up-regulated**	**Down-regulated**	**Specific**	**Up-regulated**	**Down-regulated**	**Specific**
Citrate synthase I (TGME49_268890-CSI)		K117	K459(ME49)		K117	K289(RH) K543(RH)
Aconitate hydratase (TGME49_226730-ACN)		K435		K569, K1,012		
Isocitrate dehydrogenase (TGME49_266760-IDH)						K326(ME49) K498(RH)
Succinyl-CoA-synthetase alpha (TGME49_290600-SCSA)		K439		K94, K184, K543		K74(RH)
Succinate dehydrogenase (TGME49_215280-SDHA)			K308(RH)			K120(RH) K306(RH) K320(RH)
Fumarate hydratase (TGME49_267330-FH)						K601(RH) K635(RH), K422(ME49)
Malate dehydrogenase (TGME49_318430-MDH)			K490(ME49)			
Glutamate/leucine/phenylalanine/ valine dehydrogenase family protein (TGME49_249390-GDH)	K566		K304(ME49)	K283, K417, K576, K850		K847(RH)
NADP-specific glutamate dehydrogenase (TGME49_293180)				K323	K312	K354(RH)
Catalase (TGME49_232250-CAT)						K222(ME49) K240(RH)
Enoyl-CoA hydratase/isomerase family protein (TGME49_224090-ECH)		K439				K513(RH)
(RH) represents a specific modification site of *T. gondii* RH strain. (ME49) represents a specific modification site of *T. gondii* ME49 strain (detailed data are listed in Supplementary Data 20).						

Table S4: The differentially crotonylated and 2-hydroxyisobutyrylated proteins related to aminoacyl-tRNA biosynthesis

		Crotonylation			2-hydroxyisobutyrylation		
Types	Gene	Up-regulated	Down-regulated	Specific	Up-regulated	Down-regulated	Specific
Alanine,aspartate and glutarnate metabolism	glutamate-tRNA ligase		K235		K455		
	glutaminyl-tRNA synthetase (GlnRS)		K575, K753			K569	K65(ME49)<br>K700(RH)
	cytosolic tRNA-Ala synthetase			K545(RH)<br>K551(ME49)		K516, K551, K788	K640(ME49)<br>K780(RH)<br>K1,146(RH)
	asparaginyl-tRNA synthetase				K257, K540	K620	K376(RH)
Glycine,seine and threonine metabolism	glycyl-tRNA synthetase				K402		K410(RH)
	threonyl-tRNA synthetase family protein			K383(RH)	K797	K618	
	phosphoseryl-tRNA (Sec) selenium transferase					K4,531	
Cysteine and methionine metabolism	cysteine-tRNA synthetase (CysRS)				K656	K264	
	methionyl-tRNA synthetase				K424		
Valline,leucine and isoleucine biosynthesis	valyl-tRNA synthetase				K1,002, K1,083	K1,101	K382(RH) K641(RH) K667(RH) K1,061(RH)
	leucyl-tRNA synthetase				K575	K906	
	isoleucyl-tRNA synthetase family protein					K295, K605	K456(RH)
Lysine biosynthesis	lysine-tRNA ligase			K379(RH)	K575		K138(RH)
Arginine and proline metabolism	arginyl-tRNA synthetase family protein				K214, K289		
	Prolyl-tRNA synthetase (ProRS)		K533	K581(RH)	K269, K387, K638	K254, K525, K648	
Tryptophan metabolism	Tryptophanyl-tRNA synthetase (TrpRS2)	K222	K565				

(RH) represents a specific modification site of *T. gondii* RH strain. (ME49) represents a specific modification site of *T. gondii* ME49 strain.
Cytosolic tRNA-Ala synthetase (TGME49_219540-6.1.1.7), tryptophanyl-tRNA synthetase (TrpRS2) (TGME49_288360-6.1.1.2), glutaminyl-tRNA synthetase (GlnRS) (TGME49_217460-6.1.1.18), prolyl-tRNA synthetase (ProRS) (TGME49_219850-6.1.1.15), glutamate-tRNA ligase (TG_ME49_263870-6.1.1.17), threonyl- tRNA synthetase family protein (TGME49_300260-6.1.1.3) and lysine-tRNA ligase (TGME49_205710-6.1.1.6), asparaginyl-tRNA synthetase (TGME49_270510-6.1.1.22), glycyl-tRNA synthetase (TGME49_256990-6.1.1.14), phosphoseryl-tRNA (Sec) selenium transferase (TGME49_315170-2.9.1.2), cysteine-tRNA synthetase (CysRS) (TGME49_299810-6.1.1.16), methionyl-tRNA synthetase (TGME49_289300-6.1.1.10), leucyl-tRNA synthetase (TGME49_292080-6.1.1.4), valyl-tRNA synthetase (TGME49_253290-6.1.1.9), isoleucyl-tRNA synthetase family protein (TGME49_207640-6.1.1.5), arginyl-tRNA synthetase family protein (TGME49_210840-6.1.1.19) (detailed data are listed in Supplementary Data 21).

Table S5: The differentially crotonylated and 2-hydroxyisobutyrylated proteins involved in protein biosynthesis, folding, and ubiquitin-dependent degradation

	Crotonylation			2-hydroxyisobutyrylation		
**Type**	**Up-regulated**	**Down-regulated**	**Specific**	**Up-regulated**	**Down-regulated**	**Specific**
**heat shock protein**						
HSP20			K116(RH)		K113	K58(RH)
HSP28						K242(ME49)
HSP29				K187		K186(RH)
HSP60			K417(RH)			K190(RH)
HSP70	K113, K597	K509	K253(RH)	K129, K599, K253, K591, K528	K93, K574	
HSP90				K524, K536		K250(RH), K269(RH)
**ubiquitin**						
ubiquitin-conjugating enzyme subfamily protein				619	K625	
ubiquitin family protein				K33		
ubiquitin carboxyl-terminal hydrolase UCHL3					K144	
ubiquitin-activating enzyme E1 family						K356(RH), K443(RH), K902(RH)
**proteasome**						
26S proteasome regulatory subunit						K25(RH), K332(RH)
proteasome 26S regulatory subunit				K336		K49(RH), K328(RH)
ubiquitin carboxyl-terminal hydrolase						K143(RH), K291(RH)

(RH) represents a specific modification site of *T. gondii* RH strain. (ME49) represents a specific modification site of *T. gondii* ME49 strain (detailed data are listed in Supplementary Data 21).

Table S6: Proteomic studies of PTMs in *T. gondii*

Strain	Parasite stage	PTM types	Proteins	PTM sites	Proteome Coverage (%)	Function	Reference
RH	Intracellular	Lysine acetylation	274	411	3.3%	transcription, translation, metabolism, and stress responses	(Jeffers and Sullivan *et al*., 2012)
RH	Extracellular	Lysine acetylation	386	571	4.6%	metabolism, translation, and chromatin biology	(Xue *et al*., 2013)
RH	Intra-and extracellular	Arginine Methylation	370	618	4.5%	transcriptional regulation and splicing biology	(Yakubu, *et al*., 2017)
GT1	Extracellular	O-GlcNAcylation				cell cycle, intracellular transport of proteins, protein turnover,	(Perez-Cervera,Y *et al*., 2011)
RH	Intracellular	Cysteine palmitoylation myristoylation, prenylation	401		4.8%	metabolic processes, transcription and translation, gliding and host-cell invasion	(Caballero *et al*., 2016)
RH	Intracellular	Cysteine palmitoylation	282		3.4%	invasion, motility, and cell morphology	(Foe *et al*., 2015)
T. gondii		Cysteine palmitoylation	112			host cell invasion, parasite motility and organelle biogenesis	(Frenal *et al*.,2013)
RH	Intra-and extracellular	Ubiquitination	454	800	5.4%	cell division and cell cycle	(Silmon de Monerri *et al*., 2015)
RH	Extracellular	SUMO	120		1.4%	host cell invasion and cyst genesis	(Braun *et al*., 2009)
RH	Extracellular tachyzoites	Lysine succinylation	147	425	1.8%	metabolism, epigenetic gene regulation	(Li *et al*., 2014)
RH	Intracellular	Phosphorylation	892	1,619	10.6%	host cell signaling activation/deactivation	(He *et al*., 2017)
RH	Intracellular<br>Intracellular (Purified)	Phosphorylation	2793<br>3506	12793<br>24298	33.2%<br>41.64%	host-pathogen interaction	(Treeck *et al*.,2011)
RH	Extracellular	Lysine crotonylation	1,061	3,735	12.6%	ribosome, proteasome, pentose	

						phosphate pathway, microbial metabolism in diverse environments, glycolysis/ gluconeogenesis, citrate cycle (TCA cycle), Aminoacyl−tRNA biosynthesis, carbon metabolism, Biosynthesis of amino acids, peroxisome, carbon fixation in photosynthetic organisms, gap junction, 2-oxocarboxylic acid metabolism, glutathione metabolism, MAPK signaling pathway, ferroptosis,	
ME49	Extracellular	Lysine crotonylation	984	3,396	1.7%	ribosome, proteasome, pentose phosphate pathway, microbial metabolism in diverse environments, citrate cycle (TCA cycle), carbon metabolism, aminoacyl−tRNA biosynthesis, RNA transport, glycolysis/gluconeogenesis, oxidative phosphorylation	
RH	Extracellular	Lysine 2-hydroxyisobutyrylation	1,950	9,502	23.2%	ribosome, proteasome, glycolysis / gluconeogenesis, Citrate cycle (TCA cycle), Carbon metabolism, Biosynthesis of amino acids, Carbon fixation in photosynthetic organisms, spliceosome, peroxisome	
ME49	Extracellular	Lysine 2-hydroxyisobutyrylation	1720	8,092	20.5%	Ribosome, Proteasome, Glycolysis / Gluconeogenesis, Citrate cycle (TCA cycle), Carbon metabolism, Aminoacyl−tRNA biosynthesis, Carbon fixation in photosynthetic organisms, Alanine, aspartate and glutamate metabolism	

Table S7: Detected modification sites on *T. gondii* histones

Histone	Crotonylation (K)	2-hydroxyisobutyrylation (K)	Ubiquitin (K) (RH)	Succinyl (K) (RH)	Acetyl (K) (RH)
H2Bv	105,117	9,19,43,53,54,105,113,117	14,15,19,40,43,113,117	None	9,14,15,19
H4	68	13,32,68,80 (RH)	32,60,68	32	13,17,32
H3	57	24,28,57,80,123	24,28,57,116	57,123	10,15,19,24,80
H3.3	57	57	None	None	57*
H2A1	74,149	74,149	74,149,172,173	None	None
H2Ax	98(RH)	23,128 (RH)	23,124,126,128	None	128
H2Az	6(ME49),10(RH),18,124	6,10,18,24,124,143	10,18,24,27,143,151,152	None	10,14,18,24,27,29
H2Ba	35,38,71(RH),100,112	35(RH),38,48,71,78(RH),100,108,112	77,78,100,108,112,116	38,71,100,108	35*,100*,112*
H2Bb	None	70	None	None	35*,100*,112*
* An asterisk indicates that due to high homology between some histone variants, it was not possible to discern if these acetyl marks are present on one or both of the histones listed. (RH) represents a specific modification site of *T. gondii* RH strain. (ME49) represents a specific modification site of *T. gondii* ME49 strain.					
